# Profound genetic divergence and asymmetric parental genome contributions as hallmarks of hybrid speciation in polyploid toads

**DOI:** 10.1098/rspb.2017.2667

**Published:** 2018-02-07

**Authors:** Caroline Betto-Colliard, Sylvia Hofmann, Roberto Sermier, Nicolas Perrin, Matthias Stöck

**Affiliations:** 1 Department of Ecology and Evolution, University of Lausanne, Biophore Building, 1015 Lausanne, Switzerland; 2 Department of Conservation Biology, UFZ Helmholtz-Centre for Environmental Research, Permoserstrasse 15, 04318 Leipzig, Germany; 3 Leibniz-Institute of Freshwater Ecology and Inland Fisheries (IGB), Müggelseedamm 301, 12587 Berlin, Germany

**Keywords:** polyploidy, hybridization, divergence, multi-locus phylogeny, directional asymmetry, *Bufo viridis* subgroup

## Abstract

The evolutionary causes and consequences of allopolyploidization, an exceptional pathway to instant hybrid speciation, are poorly investigated in animals. In particular, when and why hybrid polyploids versus diploids are produced, and constraints on sources of paternal and maternal ancestors, remain underexplored. Using the Palearctic green toad radiation (including bisexually reproducing species of three ploidy levels) as model, we generate a range-wide multi-locus phylogeny of 15 taxa and present four new insights: (i) at least five (up to seven) distinct allotriploid and allotetraploid taxa have evolved in the Pleistocene; (ii) all maternal and paternal ancestors of hybrid polyploids stem from two deeply diverged nuclear clades (6 Mya, 3.1–9.6 Mya), with distinctly greater divergence than the parental species of diploid hybrids found at secondary contact zones; (iii) allotriploid taxa possess two conspecific genomes and a deeply diverged allospecific one, suggesting that genomic imbalance and divergence are causal for their partly clonal reproductive mode; (iv) maternal versus paternal genome contributions exhibit asymmetry, with the maternal nuclear (and mitochondrial) genome of polyploids always coming from the same clade, and the paternal genome from the other. We compare our findings with similar patterns in diploid/polyploid vertebrates, and suggest deep ancestral divergence as a precondition for successful allopolyploidization.

## 1. Introduction

How much hybridization and introgression events contribute to speciation and genome evolution is developing as an active research topic [1,2]. At least in plants (e.g. [2–5]), polyploid hybrid speciation appears more common than homoploid hybrid speciation. This question has been less investigated in animals, due to both lower incidence of polyploid hybrid speciation and smaller economic importance (cf. [6,7]). Research efforts in amphibians have mainly involved cytogenetics (overview: [6,8]). Advanced recent molecular approaches, allowing dating and genome-wide evidence, have been applied to Pipidae (e.g. [9–12,13]) and Ambystomatidae [14–17]. Nevertheless, important questions regarding hybrid speciation remain to be addressed, such as: what circumstances favour allopolyploid over diploid hybrid formation? And: what specific constraints govern allopolyploid formation, in terms of origin and differentiation of paternal and maternal genomes?

The divergence of parental lineages is expected to affect opportunities for hybrid speciation and allopolyploid formation [18]. Reproductive isolation between diploid lineages, and thus introgression, tends to scale with divergence [19–22], with complex effects on hybrid meiosis. In particular, hybridization between genetically similar lineages presents higher opportunities for multi-valent formation, mis-segregation and chromosome rearrangements during meiosis, which poses major challenges to early polyploid evolution [23,24]. By contrast, multi-valent formation is less likely if hybridizing lineages exhibit greater divergence and structural genome differentiation [23]. Accordingly, several meta-studies of hybrid plants have suggested that genetic divergence is greater for parents of polyploids than of homoploids [25] (but see [26]). Chapman & Burke [25] furthermore hypothesized that triploids arise from diploid hybrids via meiotic non-reduction (resulting in diploid gametes), followed by fertilization with haploid pollen. Thus, the production of unreduced gametes, associated with increased divergence time, has been considered as a mechanism facilitating allopolyploidization [27].

This complex relationship between divergence, meiosis and ploidy in asexual hybrids (also documented from vertebrates) has inspired the ‘balance hypothesis’ [28,29], which proposes that parental genome divergence has to be large enough to affect meiosis in hybrids (so as to produce enough unreduced gametes), but not too large to maintain some hybrid viability or fertility. The ratios of parental genomes may further affect hybrid meiosis by generating additional difficulties in AAB or ABB triploids (when compared with AABB allotetraploids), potentially leading to asexuality [29]. Extrapolating to animals Chapman & Burke's [25] suggestion, we therefore predict that (i) the parental species of polyploid hybrids should exhibit greater divergence than those involved in the formation of contact zones with variably introgressed diploid hybrids, and (ii) ameiotic hybrids should result from both profound parental divergence and unequal parental genome contributions.

In addition, hybrid and allopolyploid formation may be governed by the direction of hybridization. Reciprocal hybrids often show asymmetric fitness differences that stem from dominance effects in Dobzhansky–Muller incompatibilities [30], originating from sex chromosomal versus autosomal (e.g. Haldane's rule [31–33]) or cyto-nuclear interactions. Therefore, we further hypothesize that the evolution of allopolyploid lineages may show similar asymmetric interactions.

To test these questions in amphibians, Palearctic green toads (*Bufo viridis* subgroup) present a highly suitable system to compare diploid and polyploid hybridization within one radiation. This group comprises different diploid lineages forming secondary contact zones, with levels of introgression that scale with divergence [22,34]. Furthermore, bisexually reproducing species of three ploidy levels (2n, 3n and 4n) have been described from Central Asia [35]. Maternal ancestry has been inferred from mtDNA sequences plus nuclear microsatellites for two allopolyploids (3n *B. baturae*, 4n *B. pewzowi*) [36–38], and mtDNA only (entirely missing nuDNA evidence) for three other presumably allopolyploid species (*B. oblongus, B. pseudoraddei* and *B. zugmayeri*) [39]. Another six Eurasian diploid species have unclear nuclear relationships to the polyploids, which calls for integration into a comprehensive phylogenetic analysis. Diploid and tetraploid green toads reproduce meiotically ([39] incl. refs.), while one triploid species (*B. baturae*) has a partly ameiotic gametogenesis [40], possibly also found in two other triploid forms (systematic details: electronic supplementary material, text S1).

In this paper, we use new multi-locus nuclear sequence data, supplemented by mitochondrial DNA, to (i) identify allopolyploidization events in the Palearctic green toad radiation, (ii) infer the paternal and maternal ancestries of polyploids, (iii) compare the genetic divergence of lineages involved in diploid versus allopolyploid hybrid formation, and (iv) test for a possible directionality in hybridization events (namely, asymmetries in the origin and contribution of maternal versus paternal genomes to allopolyploid formation).

## 2. Material and methods

### (a) Animal sampling and DNA extraction

**Figure 1.**
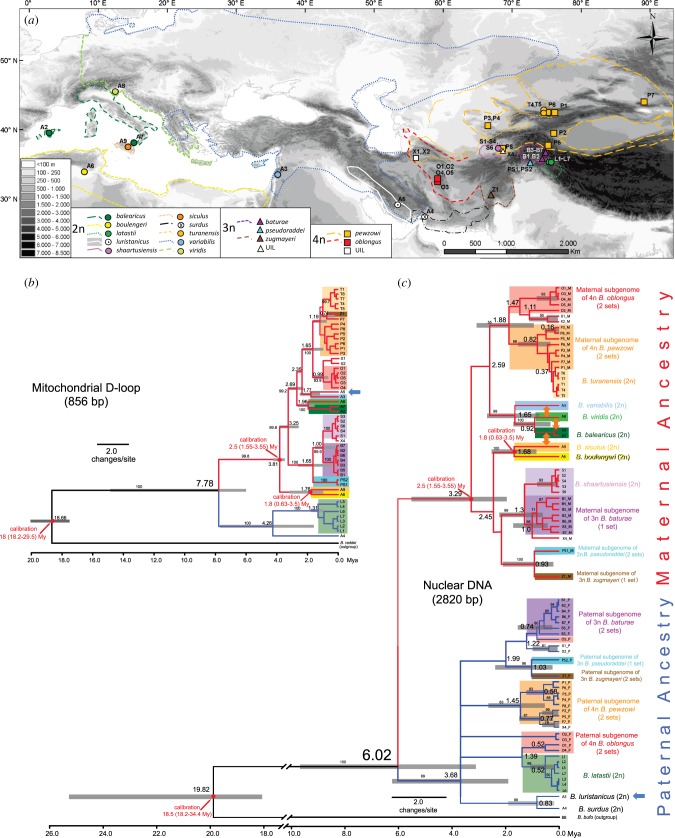
Green toads in geographical and phylogenetic context. (*a*) Map with sampling localities, approximate range borders and sample abbreviations as used in (*b,c*) and electronic supplementary material, table S1. (*b*,*c*) Bayesian trees of approximately 856 bp of the mitochondrial D-loop (*b*) and of approximately 2820 bp of concatenated nuclear DNA (*c*) as obtained with the program BEAST v. 1.8.3. Subclades shown in red (maternal ancestry) are also referred to as ‘western clade’; subclades shown in blue (paternal ancestry) are referred to as ‘eastern clade’. Orange double arrows, in (*c*) between branches, within the western clade, indicate known natural hybridization between diploid lineages including the formation of diploid/diploid hybrid zones with introgression [22,34]. Blue arrows at sample A5 point to an incongruent mitochondrial versus nuclear phylogenetic position of *B. luristanicus*. Small numbers at branches show Bayesian posterior support values (greater than 50%); large numbers at nodes and time scales below trees in (*b*) and (*c*) show divergence time estimates (mean values) in millions of years ago (Mya); grey bars in trees indicate 95% confidence intervals for nodes with sufficient posterior support. An extended version of this figure is provided in the electronic supplementary material, figure S1.

Our study includes a total of 51 green toads from scientific collections as specified (figure 1*a*; electronic supplementary material, text S2 and table S1) [35,37,39], obtained between 1997 and 2012 from 32 localities across their Palearctic range. This comprises 15 taxa from all currently known major mtDNA clades [37], as well as three taxonomically unassigned toads, namely two tetraploids (X1, X2) and one triploid (X4), further abbreviated ‘UIL’ (unidentified lineage). Samples of *B. bufo and B. raddei* were used as outgroups.

### (b) Amplification and sequencing of nuclear markers

Six nuclear sequence markers (*CYP19*, *DMRT1*, *SF-1*, *SPAG6*, *SOX3*, *VLDLR*), several of which are involved in vertebrate sexual development and differentiation, representing different linkage groups of the anuran genome were developed using orthologues on *Xenopus tropicalis* scaffolds 1, 3, 6, 8 and 19. Primers for cross-amplifying markers (electronic supplementary material, Text S2, and table S2) were designed using a *B. viridis* transcriptome (GenBank Biosample SAMN03993917 [41]). Markers were PCR amplified (electronic supplementary material, texts S1 and S3). Amplicons were extracted from agarose gels, purified using the Wizard SV Gel and PCR clean-up system (Promega) and a single final amplicon pool was obtained for each individual by mixing equimolar amounts of these products. Each individual pool was barcoded prior to further pooling of all 48 mixes, which were NGS-sequenced using the Roche/454 GS-FLX Titanium platform by LGC Genomics Corporation (UK) with a coverage of greater than 80× per PCR product. Alleles were then screened and edited manually to eliminate singletons, and contigs with greater than 15× coverage considered a true allele; the maximum number of alleles was inferred according to ploidy (2 in 2n, 3 in 3n, 4 in 4n). To complete the dataset for this radiation with three initially unconsidered polyploid species, for three samples exclusively (*B. zugmayeri* (Z1)*, B. siculus* (A9) and *B. pseudoraddei* (PS2)), nuclear PCR products were cloned using the TOPO TA cloning kit (with pCR II-TOPO-vector system; Invitrogen) according to the manufacturer's protocol. To detect heterozygotes, at least 12 clones were Sanger-sequenced in diploids and 24 in triploids, edited to eliminate singletons, and added to the rest of the dataset.

### (c) Sequencing and phylogenetic analyses of mtDNA

The mitochondrial control region (D-loop, approx. 880 bp) was amplified as described [37,42]. Products were Sanger-sequenced in both directions and contigs edited in Sequencher v. 4.9. Bayesian phylogenetic analyses were carried out with MrBayes v. 3.2.6 [43] using the best-fit model of sequence evolution (HKY + G, Bayesian information crit., BIC) as determined by jModeltest v. 2.1.7 [44] (electronic supplementary material, text S2). Stationarity and convergence of the runs were confirmed using the software Tracer v. 1.7.2 [45]. The first 25% of each run was discarded as burn-in.

### (d) Subgenome inference and phylogenetic analyses of nuclear markers

Nuclear DNA sequences, in total 2820 bp, were aligned using ClustalW multiple alignment in BIOEDIT [46]. For each marker, a maximum-likelihood phylogenetic analysis was performed using PhyML (v. 3.0; [47]). The maternal ancestor of each allopolyploid was assigned according to mtDNA haplotype, from which the maternal (and by deduction, paternal) subgenomes could be inferred based on microsatellite allelic range similarity (in part identical samples as in [36]). To allow proper concatenation of ancestral nuclear markers, we retained only one consensus sequence from heterozygous fragments of diploid individuals, replacing SNPs with ‘Ns’ (i.e. any base). The same was done in allopolyploids, from which two consensus sequences were kept, corresponding to their inferred maternal and paternal subgenomes, respectively. Finally, all resulting nuclear sequences were concatenated in the 5′–3′ direction to obtain a ‘super-alignment’ for phylogenetic analyses (electronic supplementary material, text S2).

### (e) Molecular dating

Molecular dating for major lineages was performed based on the concatenated nuclear dataset, and separately for mtDNA, using the Bayesian relaxed-clock approach as implemented in BEAST v. 1.8.3 [48]. We determined the most suitable substitution models per partition (nuDNA), using PartitionFinder v. 1.1.1 [49] or for the entire mtDNA marker, using jModeltest v. 2.1.7 [44]; divergence time analyses were run with substitution models unlinked between partitions. We included an outgroup for both nuDNA and mtDNA, and imposed three available age constraints to the molecular clock (electronic supplementary material, text S2).

We generated a random starting tree and assumed an uncorrelated lognormal relaxed molecular clock and a Yule process as a model of speciation, as this prior is most appropriate for species-level divergences [48]. Two independent runs were performed with 200 million generations, sampling 10 000 trees and with a burn-in set to 25% of the samples. Convergence and stationary levels were verified with Tracer v. 1.7.2. We annotated the tree information with TreeAnnotator v. 2.3.1 and visualized it with FigTree v. 1.4.2 [48]. All runs were performed on the CIPRES Science Gateway [50].

## 3. Results

### (a) Phylogenetic analyses of mitochondrial DNA

Bayesian analysis of maternally inherited mitochondrial DNA resulted in distinct haplotypes and clades that mostly coincide with the previously distinguished nominal taxa. This analysis unveiled a very deep divergence between *B. surdus* and *B. latastii* on one side (figure 1*b*, blue-marked clade, hereafter ‘eastern’; electronic supplementary material, figures S1 and S2) and the remaining taxa on the other side (red-marked clade, hereafter ‘western’), with an estimated divergence of 7.7 (4.4–12.7) Mya. Interestingly, several tetraploid (*B. pewzowi*, *B. oblongus*, plus X1 and X2) and one triploid species (*B. zugmayeri*) share a Pleistocene (1.65 (0.9–2.5) Mya) mitochondrial ancestor with the diploid *B. turanensis*, while other triploids (*B. baturae, B. pseudoraddei* and X4) share a similarly old Pleistocene (1.65 (0.72–2.76 Mya)) mtDNA-ancestry with a different diploid species (*B. shaartusiensis*). We further note that the diploid *B. turanensis* does not take a basal position in its subclade but appears to be derived from the polyploids (electronic supplementary material, text S4).

### (b) Phylogenetic analyses of nuclear DNA

Phylogenies obtained from single genes are shown in electronic supplementary material, figures S3–S8. Allele numbers (haplotypes) therein varied between 1 and 6. The analyses of the concatenated sequences (2820 bp) yielded two highly supported clades (red and blue, figure 1*c*), which diverged about 6 Mya (95% HDP, 3.1–9.7 Mya). Surprisingly, all inferred maternal genomes of the polyploid species were assigned to the ‘western clade’ (red), and all paternal genomes to the ‘eastern clade’ (blue).

The western clade split about 3.29 (1.9–5.5) Mya into two major subclades. One contains a group comprising the diploid *B. turanensis* and the maternal ancestor of the allotetraploids (*B. pewzowi* and *B. oblongus*; plus UIL X1, X2), and is itself sister to several Eurasian diploid species. The other subclade constitutes a group formed by the Asian diploid *B. shaartusiensis* and the maternal ancestor of the Asian triploid *B. baturae* (plus UIL X4), itself sister to the maternal ancestor of the two other triploids (*B. zugmayeri, B. pseudoraddei*). Many Eurasian diploid lineages from this western clade are involved in diploid hybridization across secondary contact zones in Europe [22,34] (indicated by orange arrows in figure 1*c*; electronic supplementary material, figure S1c).

The eastern clade (figure 1*c*, blue) forms a large polytomy that split about 3.7 Mya (1.9–6.3), separating a clade of diploid species (*B. surdus, B. luristanicus*) from another diploid (*B. latastii*), and containing all the paternal subgenomes of allotriploid and allotetraploid species. The paternal subgenomes of the tetraploid *B. oblongus* are split among several subclades.

The topology and divergence-time estimates for the nuclear phylogeny largely agree with the mitochondrial tree, except for the diploid *B. luristanicus*, which appears as a weakly supported sister of *B. variabilis* in the mitochondrial phylogeny (figure 1*b*) but as a sister taxon of *B. surdus* in the nuclear phylogeny (figure 1*c*). This suggests a mitochondrial capture event by the lineage of *B. luristanicus*, possibly from the partly sympatric *B. variabilis*.

## 4. Discussion

The discovery of polyploid green toads in 1976 [51] was followed by initial studies of polyploidy origins using allozymes [52] and microsatellites [36]. Here, we extend these studies through the first phylogeny of this complex based on multi-locus mtDNA and nuclear sequences, providing insights into the relative ages and contributions of maternal and paternal ancestors to allopolyploidization.

### (a) Allopolyploid origins and genome phylogenies

Our phylogenetic analysis highlighted at least five events of allopolyploidization that led to the evolution of two allotetraploids (*B. pewzowi, B. oblongus*) and three allotriploids (*B. baturae, B. pseudoraddei, B. zugmayeri*; figure 1*c*; electronic supplementary material, figure S2 (I–V) and text S5). Three additional allopolyploid forms (UIL X1, X2, X4) were also identified and characterized (figure 1*c*; electronic supplementary material, figure S2 and text S5), possibly corresponding to yet unrecognized taxa. The number of alleles (haplotypes), which varied between 1 and 6 in single-gene trees, did not allow further inferences regarding the number of hybridizations or routes to polyploidy (electronic supplementary material, figures S3–S8).

### (b) Profoundly diverged lineages form hybrid polyploids, less diverged lineages form hybrid zones

Maternal and paternal ancestors of allopolyploid taxa (4n *B. oblongus,* 4n *B. pewzowi,* 3n *B. baturae,* 3n *B. pseudoraddei,* 3n *B. zugmayeri*) in each case belong to the relatively deeply diverged western and eastern clades (6 Mya, 3.1–9.6 Mya; figure 1*c*). Divergence times distinctly exceed the much younger ones (1.9–2.6 Mya) between diploid lineages that form hybrids at secondary contacts [22,34]. Thus, despite uncertainties inherent to the calibration procedure, our phylogenies are consistent with the hypothesis that ancestors of allopolyploids exhibit greater divergence than lineages that form diploid–diploid hybrid zones with various degrees of introgression [22,34]. Our results are in line with those from plants [25] and other vertebrate allopolyploids in which parental lineages have been shown to stem from deeply diverged ancestral lineages (e.g. *Aspidocelis* and *Darevskia* lizards [53–55]; *Pelophylax* [56–58]; *Cobitis* [59]; *Squalius* [60]). This suggests that allopolyploidization might occasionally overcome the decrease in hybrid fitness resulting from the accumulation of incompatibilities with increasing divergence time.

The relative ages of within-clade diversification for maternal (and mitochondrial) and paternal ancestors of allopolyploids vary between Lower (1.8 Mya) and Mid-Pleistocene (0.93 Mya; average of 1.4 Mya; figure 1; electronic supplementary material, figure S2 and text S5). If diversification dates coincide with polyploidization events, these ages suggest that such events were triggered by Pleistocene climatic oscillations, as supported by higher resistance of polyploids to climatic stresses [61]. Ficetola & Stöck [61] have also shown that allopolyploidization might be facilitated by occupation of transgressive ecological niches, unavailable to some of the parental species.

### (c) Deeply diverged but unequal genome contributions in ameiotic forms

Whereas diploids and balanced allotetraploids reproduce by meiosis [39], ameiotic allotriploids show an unequal genomic configuration, comprising two conspecific heterozygous genomes (AA′) and a highly diverged clonal allospecific one (B) (figure 1*c*). In line with the balance hypothesis, this suggests that genomic imbalance and divergence are causal for their reproductive mode [28,29].

### (d) Directional asymmetry in parental genome contributions to allopolyploidization

The five Pleistocene events (electronic supplementary material, figure S2 (I–V)) that led to allopolyploid species formation, as well as several possibly more recent events that produced allopolyploid hybrids with unclear taxonomic status, were all unidirectional in relation to maternal and paternal ancestors. Two allotetraploids (*B. pewzowi*, *B. oblongus*; as well as UIL X1 and X2) share nuclear maternal ancestors with the same diploid (*B. turanensis*), whereas the maternal ancestry of three allotriploids (*B. baturae*, *B. zugmayeri, B. pseudoraddei*; as well as UIL X4) can be traced to another diploid (*B. shaartusiensis*; figure 1*c*); all of these belong to the same major western clade. By contrast, the entire paternal ancestry goes back to one lineage (or several related and possibly extinct lineages), represented by a single extant diploid (*B. latastii*; figure 1*c*) from the eastern clade. Moreover, in a diploid–tetraploid contact zone (*B. turanensis* and *B. pewzowi*), adult triploid F_1_-hybrids mostly have diploid *B. turanensis* as their close maternal (mtDNA) ancestor and tetraploid *B. pewzowi* as paternal ancestor [35]. These shared patterns of directional asymmetry in hybridization point to strong evolutionary constraints during allopolyploidization in green toads.

Asymmetric contributions of paternal and maternal parents are known from homoploid hybrid plants (e.g. [62]), invertebrates (e.g. [63,64]) and vertebrates (e.g. [65,66]), including interspecies crosses in bufonid toads (e.g. [67,68]). However, asymmetries have rarely been documented in allopolypoid speciation. In plants, allopolyploid origins exhibit great diversity [69–75] with few examples of asymmetric ancestry in a whole complex [76]. In vertebrates, asymmetric genome contributions to allopolyploidization remain underexplored. Tetraploids forming the *Hyla versicolor* complex originated multiple times from extant diploid *H. chrysoscelis* and two apparently extinct lineages [77], but asymmetry has not been examined. This similarly applies to the *Phyllomedusa burmeisteri* complex [78,79]. In clawed frogs, *Silurana* comprises the diploid *Silurana tropicalis* and three derived tetraploid species; *Xenopus* includes 20 described species: 11 tetraploids, 7 octoploids and 2 dodecaploids [11,13]. Several ancestral diploid species (some extinct) are maternal genome donors for some allopolyploids and paternal donors for others (cf. [9]), thus contrasting with our results. A few other systems, however, call for further exploration of possible asymmetries in genome contributions. In the *Pelophylax esculentus* complex, allodiploid (*P. esculentus, P. grafi, P. hispanica*) and allotriploid (3n *P. esculentus* with either two *P. lessonae*, RLL, or two *P. ridibundus* genomes, RRL) gonochoristic hybrids perform multi-directional genetic interactions (among themselves and with diploid parental species), blurring potential signatures of asymmetric genome contributions [56,80]. In the largely unisexual *Ambystoma jeffersonianum/A. laterale* complex, all unisexual di- and polyploid hybrids derive their mtDNA from the diploid *A. barbouri*, from which all five nuclear unisexual species diverged 2.4–3.9 Mya [17], suggesting a ‘*laterale-*like’ asymmetric maternal contribution and various paternal contributions from other bisexual species. Allodiploid, triploid and tetraploids of the mostly all-female cyprinid *Squalius alburnoides* hybrid complex exhibit multiple polyploid origins and genetic interactions, while mtDNA asymmetrically stems from the common maternal ancestor (*S. pyrenaicus*), although with rare introgression [81,82]. Similarly, in *Cobitis* loaches, multiple all-female gynogenetic allodiploid and allopolyploid hybrid lineages (with few exceptions [83]) share *Cobitis elongatoides* mtDNA, and thus a maternal nuclear ancestor [84] with Miocene divergence from the paternal ancestors (greater than 7 Mya (3.83–10.28) [59]). Several other gynogenetic and polyploid teleost complexes (for a review: [85]) are often dominated by a common maternal (mitochondrial and thereby inferred nuclear) lineage; however, gynogenetic reproduction and possible stepwise ploidy elevation complicate evaluation of potential asymmetry. These examples from few allopolyploid vertebrate complexes show several similarities to our findings and suggest that asymmetric ancestry should be more carefully addressed by future research.

Asymmetric homoploid hybridization has been explained by imbalanced barriers to gene flow under pre- or post-zygotic isolation [62,65]. Pre-mating isolation in animals is attributed to mate choice behaviours, evolved in response to sexual selection [65]. Asymmetry in post-mating isolation often results from Dobzhansky–Muller incompatibilities that involve uniparentally inherited genetic factors, such as sex chromosomes, mitochondria, epigenetic programming or maternal effects (Darwin's corollary to Haldane's rule [68,86]). Alleles involved in hybrid incompatibilities are considered partly recessive, and those on sex chromosomes are more likely expressed in the heterogametic sex [31,86]. However, asymmetric dominance in allopolyploidization has not been investigated. As the dominance model of Haldane's rule assumes degenerated sex chromosomes, whereas those in green toads are homomorphic ([39] incl. refs) [87], nuclear–cytoplasmic incompatibilities may better explain directional asymmetry under allopolyploidization in this instance.

## 5. Conclusion

Our data provide four new major insights. First, we document at least five hybridization events (up to seven; electronic supplementary material, figure S2 and text S5) that resulted in the evolution of allopolyploid species. Second, molecular dating, based on mtDNA and nuDNA, shows that allopolyploid green toads presumably originated in the Pleistocene, from ancestors that had diverged in the Miocene to Pliocene period (6 Mya (3.1–9.6 Mya); i.e. much earlier than the parents of diploid hybrids forming at secondary contacts within the western clade [22,34]). This supports the hypothesis that allopolyploidization is facilitated by greater genomic divergence. Third, we note that allotriploid ameiotic taxa always possess two conspecific genomes and a deeply diverged allospecific clonal one, suggesting that genomic imbalance and divergence are causal. Fourth, we provide evidence for directional asymmetry in maternal versus paternal genome contributions, with the maternal nuclear (and mitochondrial) genome always coming from one phylogenetic clade, and the paternal nuclear genome from the other.

This first dated nuclear phylogeny of Palearctic green toads offers new research avenues. Studies could be undertaken of sex determination in diploid ancestral versus allopolyploid derived species (cf. [87]) and whether asymmetry may not only be reflected in nuclear genome contributions but also in subgenome evolution after hybridization. This has been shown for African clawed frogs, *Xenopus laevis,* ‘with one chromosome set more often preserving ancestral states while the other experienced more gene loss, deletion, rearrangement, and reduced gene expression’ [12].

## Supplementary Material

Texts S1 to S5 and Figs. S1 to S8

## Data accessibility

Sequences have been deposited on GenBank and edited alignments have been archived on Dryad (doi:10.5061/dryad.7ns38).
