# Supplementary material for: Profound genetic divergence and asymmetric parental genome contributions as hallmarks of hybrid speciation in polyploid toads
Source: Proc Biol Sci. 2018 Feb 7;285(1872):20172667. doi: 10.1098/rspb.2017.2667 (PMC5829204; doi:10.1098/rspb.2017.2667)
Supplement: Texts S1 to S5 and Figs. S1 to S8 [file rspb20172667supp1.pdf]

<u>**Electronic Supplementary Materials (ESM):**</u>

Profound genetic divergence and asymmetric parental genome contributions as hallmarks of hybrid speciation in polyploid toads

Caroline Betto-Colliard, Sylvia Hofmann, Roberto Sermier,
Nicolas Perrin, Matthias Stöck

Supplementary Texts

Text S1: Extended Introduction and Systematic Overview 2
Text S2: Extended Methods (including **Tables S1** and **S2**) 3
Text S3: Amplification success of the nuclear markers and sequence number per species 7
Text S4: Maximum likelihood test on the placement of *B. turanensis* in the maternal clade of the Bayesian tree shown in Fig. 1c (including **Table S3)** 8
Text S5: Details on the five allopolyploidization events (**Fig. S2**: I-V). 9

Supplementary Figures

Fig. S1 Extended version of Figure 1; see legend there.
Photos: Matthias Stöck; except: *B. surdus*: Mehregan Ebrahimi, *B. variabilis*: Philip de Pous, and *B. luristanicus*: Sebastian Voitel. 14
Fig. S2: Scheme of the hybridization events (I-VII) as revealed by the present study 15
Figs. S3 to S8: Maximum likelihood phylogenetic trees for the six single nuclear markers:
Fig. S3: *CYP19* 16
Fig. S4: *DMRT1* 17
Fig. S5: *SF-1* 18
Fig. S6: *SOX3* 19
Fig. S7: *SPAG6* 20
Fig. S8: *VLDLR* 21

Text S1: Extended Introduction and Systematic Overview

Palearctic green toads (*Bufo viridis* subgroup) present a highly suitable anuran system to compare diploid and polyploid hybridization within one radiation. They comprise diploid lineages, arisen in different geological periods, forming secondary Eurasian contact zones, for which we have shown [1,2] that introgression indeed scales with divergence. In Central Asia these toads have several times evolved bisexually reproducing species of three ploidy levels (2n, 3n, and 4n; [3]), comprising meiotic and ameiotic forms. Using nuclear microsatellites without molecular dating, we have inferred the origins of two allopolyploids (*Bufo baturae* and *B. pewzowi*; [34]). Allotetraploid *B. pewzowi* (Uzbekistan to W-Mongolia) exhibits close mtDNA affinities and two inferred maternal chromosome sets to diploid *B. turanensis* [3-5] (E-Iran to S-Kazakhstan). In allo-triploid *B. baturae* (Karakoram, Hindukush, High Pamirs) mtDNA and microsatellites maternally link one chromosome set to diploid *B. shaartusiensis* (S-Tajikistan) [4,6]. The two paternal chromosome sets in both *B. baturae* and *B. pewzowi* putatively originated from a non-sampled (perhaps extinct) species, remotely related to *B. latastii* [4], representing the only known living diploid in the paternal ancestry. However, ancestry inference in *B. baturae* and *B. pewzowi* still requires nuclear DNA sequence evidence and dating. For three other, presumably allopolyploid species (*B. oblongus, B. pseudoraddei* and *B. zugmayeri*), maternal origins were suggested through mtDNA analyses [5] but nuclear DNA evidence is missing. Without this, however, mitochondrial capture (e.g. [7]), cannot be excluded. Allotetraploid *B. oblongus* (NE-Iran, W-Turkmenistan) and triploid *B. zugmayeri* (N-Baluchistan) closely relate mtDNA to *B. turanensis* [5], while *B. pseudoraddei* (all-triploid, W-Himalaya) forms a mitochondrial sister to *B. baturae* [5]. Importantly, the paternal ancestors of all three species have remained unknown.

Analyses of hybrid origins should include the entire radiation of Palearctic green toads, which further includes the diploid lineages *B. boulengeri* (North-Africa), *B. siculus* (Sicily), *B. balearicus* (Italy, West-Mediterranean islands), *B. luristanicus* (SW-Iran), *B. surdus* (S- and SE-Iran), *B. variabilis* (Asia Minor, E-Europe and N-Central Asia) and *B. viridis* (Central and E-Europe). All diploid and tetraploid green toads reproduce meiotically ([8] incl. refs.), while triploid *B. baturae* reproduces by 'pre-equalizing hybrid meiosis' [9], where males produce haploid sperm, while females simultaneously transmit a clonal, and a recombined chromosome set [9]; a mechanism that is presumbaly also occurring in all-triploid *B. pseudoraddei* and *B. zugmayeri*.

Text S2: Extended Methods

Details on animal sampling and DNA extraction

Adult individuals were documented photographically and sampled for buccal cells [10], fingertips or tail tips (tadpoles) before release or deposit in scientific collections (Table S1). Swabs and tissue samples (in 70% ethanol) were stored at -20°C. DNA was extracted using the DNeasy Tissue Kit or the BioSprint robotic workstation (QIAGEN), following the manufacturer's protocols. DNA was eluted in volumes of 150 µl and 50 µl (QIAGEN Buffer AE) and stored at -20°C.

Details on amplification and sequencing of nuclear markers

Polymerase chain reactions (PCR) in 10 µL-reactions, containing 1 µL DNA (25 ng/µL), 1 µL 10x PCR Buffer (15 mM $MgCl_2$), 1 µL dNTPs (2.5 mM each), 0.5 µL each primer (10 µM), and marker specific volumes of $MgCl_2$ and QIAGEN Taq (5 U/µL; (details: Table S2). Amplifications of all markers (except *VLDLR*) were carried out using the thermal profile: 95°C, 3 min, initial denaturation: [95°C, 30 s; T_A, 30 s; 72°C, 30 s] x cycles; 72°C, 5 min, final elongation (Table S2). The *VLDLR* gene fragment was amplified by touch-down PCR: 95°C, 3 min, initial denaturation; [95°C, 30 s; decreasing annealing temperature from 54°C to 48°C of -1°C per cycle, 30 s; 72°C, 30 s] x 7 cycles; followed by [95°C, 30 s; 48°C, 30 s; 72°C, 30 s] x 33 cycles; 72°C, 5 min, final elongation. We used the GeneAmp PCR System 2700 and 9700 (Perkin Elmer).

Details on phylogenetic analyses of mtDNA

We performed four runs with 20 million generations with four chains, starting with a random tree and sampling every 2000 generations, until reaching an average standard deviation of split frequencies of < 0.01. Stationarity and convergence of the runs were confirmed using the software Tracer v.1.7.2 (http://beast.bio.ed.ac.uk/Tracer). The first 25% of each run were discarded as burn-in.

Details on subgenome inference and phylogenetic analyses of single nuclear markers

The best-fitting model of marker-specific sequence evolution was selected (Akaike information criterion, AIC; Table S2). PhyML (ver. 3.0) was used with the SPR branch swapping algorithm and, to assess node support, with 10^3 bootstrap replicates (continued on next page). We then generated maximum likelihood-based phylogenies using PhyML (ver. 3.0, [11]) with a GTR + Γ +I model of sequence evolution, the SPR branch swapping algorithm and 10^3 bootstrap replicates. The phylogenetic hypothesis was visualized using FigTree (v.1.4.2; http://tree.bio.ed.ac.uk/software/figtree/).

Sample ID	Field No	Voucher	Species	Ploidy	Sex / Type	Locality	Coordinates
L1	SK1xSK2_40	-	*Bufo latastii*	2n	tadpole	Skardu, Pakistan	35.3 N, 75.37 E
L2	SK3xSK4_60	-	*Bufo latastii*	2n	tadpole	Skardu, Pakistan	35.3 N, 75.37 E
L3	SK7xSK8_40	-	*Bufo latastii*	2n	tadpole	Skardu, Pakistan	35.3 N, 75.37 E
L4	Bufo 128	-	*Bufo latastii*	2n	juvenile	Satpara River, SW of Skardu, Pakistan	35.283 N, 75.617 E
L5	SK17xSK18_40	-	*Bufo latastii*	2n	tadpole	Skardu, Pakistan	35.3 N, 75.37 E
L6	SK9xSK10_1	-	*Bufo latastii*	2n	tadpole	Skardu, Pakistan	35.3 N, 75.37 E
L7	SK13x14_2	-	*Bufo latastii*	2n	tadpole	Skardu, Pakistan	35.3 N, 75.37 E
T1	Ky111	NME A 1852/12	*B. turanensis*	2n	female	S of Bishkek, Kyrgyzstan	42.792 N, 74.684 E
T4	Bufo 14	-	*B. turanensis*	2n	male	Botanical garden, Bishkek, Kyrgyzstan	42.858 N, 74.591 E
T5	Ky22	MVZ 249176	*B. turanensis*	2n	male	S of Bishkek, Kyrgyzstan	42.691 N, 74.662 E
T6	Ky15x16_4a	-	*B. turanensis*	2n	tadpole	S of Bishkek, Kyrgyzstan	42.796 N, 74.691 E
T7	Ky110a_24a	-	*B. turanensis*	2n	unknown	S of Bishkek, Kyrgyzstan	42.792 N, 74.684 E
S1	SzI_20	-	*B. shaartusiensis*	2n	tadpole	Shaartuz, Tajikistan	37.295 N, 68.133 E
S2	Sz2_20	-	*B. shaartusiensis*	2n	tadpole	Shaartuz, Tajikistan	37.295 N, 68.133 E
S3	Sz22xSz23_38	-	*B. shaartusiensis*	2n	tadpole	Shaartuz, Tajikistan	37.295 N, 68.133 E
S4	Sz10	-	*B. shaartusiensis*	2n	male	Shaartuz, Tajikistan	37.295 N, 68.133 E
S6	Sz12	-	*B. shaartusiensis*	2n	male	Shaartuz, Tajikistan	37.295 N, 68.133 E
B1	Bufo 27	ZSM 101/1998	*B. baturae*	3n	female	Sust, Pakistan	36.767 N, 74.833 E
B2	Bufo 28	ZSM 102/1998	*B. baturae*	3n	male	Sust, Pakistan	36.767 N, 74.833 E
B3	Bufo 37	ZSM 112/1998	*B. baturae*	3n	male	Gilgit, Pakistan	35.9 N, 74.4 E
B4	Bufo 36	ZSM 111/1998	*B. baturae*	3n	female	Gilgit, Pakistan	35.9 N, 74.4 E
B5	G4xG5_2	-	*B. baturae*	3n	tadpole	Gilgit, Pakistan	35.9 N, 74.4 E
B6	G7xG8_50	-	*B. baturae*	3n	tadpole	Gilgit, Pakistan	35.9 N, 74.4 E
B7	G3xG4_21	-	*B. baturae*	3n	tadpole	Gilgit, Pakistan	35.9 N, 74.4 E
P1	Bufo 11	MTD 40012	*B. pewzowi*	4n	male	Issyk-Kul, Kyrgyzstan	42.467 N, 76.200 E
P2	Bufo 22	ZSM 107/1998	*B. pewzowi*	4n	male	Kashgar, Xinjiang, China	39.483 N, 76.033 E
P3	Bufo 15	MTD 39405	*B. pewzowi*	4n	male	Nuratau-Range, Uzbekistan	40.583 N, 66.500 E
P4	Bufo 16	MTD 39406	*B. pewzowi*	4n	male	Nuratau-Range, Uzbekistan	40.583 N, 66.500 E
P5	Bufo 25	ZSM 110/1998	*B. pewzowi*	4n	female	Taxkurgan, E-Pamir, China	37.783 N, 75.233 E
P6	Ky56	MVZ 250751	*B. pewzowi*	4n	male	S of Bishkek, Kyrgyzstan	42.644 N, 74.614 E
P7	Bufo 200	CAS 171676	*B. pewzowi*	4n	male	Canyon above Dayou, S of Dayou, China	43.986 , 89.066 E
P8	Sz11	-	*B. pewzowi*	4n	male	Shaartuz, Tajikistan	37.295 N, 68.133 E
O1	Bufo 221	CAS 228693	*B. oblongus*	4n	male	S of Jaanbaazaan Square, Birjand, Iran	32.822 N, 59.218 E
O2	Bufo 222	CAS 228694	*B. oblongus*	4n	male	S of Jaanbaazaan Square, Birjand, Iran	32.822 N, 59.218 E
O3	Bufo 45	-	*B. oblongus*	4n	female	Birjand, Iran	32.550 N , 59.160 E
O4	Bufo218	CAS 228690	*B. oblongus*	4n	male	S of Jaanbaazaan Square, Birjand, Iran	32.822 N, 59.218 E
O5	Bufo220	CAS 228692	*B. oblongus*	4n	male	S of Jaanbaazaan Square, Birjand, Iran	32.822 N, 59.218 E
X1	Bufo 216	MVZ 245917	Unidentified	4n	male	Touran protected area, ~120 km ESE of Shahrud, Iran	35.967 N, 56.068 E
X2	Bufo 217	CAS 228604	Unidentified	4n	male	Touran protected area, ~120 km ESE of Shahrud, Iran	35.967 N, 56.068 E
X4	Sz14	-	Unidentified	3n	male	Shaartuz, Tajikistan	37.295 N, 68.133 E
PS1	Bufo24	ZSM 106/1998	*B. pseudoraddei pseudoraddei*	3n	female	Swat-Valley, Kulalai, Pakistan	35.320 N, 72.615 E
PS2	Bufo137	-	*B. pseudoraddei pseudoraddei*	3n	juvenile	Swat-Valley, Kulalai, Pakistan	35.320 N, 72.615 E
Z1	Bufo345	MVZ 250385	*B. zugmayeri*	3n	subadult	Pishin, Pakistan	30.580 N, 67.000 E
A2	B85	-	*B. balearicus*	2n	tadpole	Llucmajor, Mallorca, Spain	39.498 N, 2.879 E
A7	Si13	-	*B. balearicus*	2n	male	Mazzarà, Sicily, Italy	38.096 N, 15.138 E
A3	Bufo51	MTKD D 42567	*B. variabilis*	2n	male	Barqash, Syria	33.483 N, 36.0 E
A4	Bufo59	MTKD D 43944	*B. surdus*	2n	male	Deh Barez, Baluchestan, Iran	27.450 N, 57.32 E
A5	Bufo58	MTKD D 43943	*B. luristanicus*	2n	male	Posht Chenar, Frynta leg., Iran	29.20 N, 53.33 E
A6	Bufo166	MVZ 235680	*B. boulengeri*	2n	female	Tozeur, Tunisia	33.91 N, 7.98 E
A8	Bv61	-	*B. viridis*	2n	tadpole	Treporti, Italy	45.466 N 12.455 E
A9	Si334	-	*B. siculus*	2n	male	Pergusa Lake, Enna Province, 673 m a.s.l., Italy	37.517 E, 14.301 E
BB	Outgroup	-	*B. bufo*	2n	juvenile	Halle (Saale), Kreuzer Teiche, Germany	51.503 N, 11.994 E

Table S1: Sampling overview. Sample ID (as in text and Fig. 1), field number, scientific voucher number (if available), scientific name, ploidy level, sex for adult individuals or form of juvenile individuals, sampling locality description and geographic coordinates (decimal degrees) for each sample.

Marker Abbreviation	Marker name	*X. tropicalis* scaffold	Segments	Size	Primers	$MgCl_2$	Taq	T_A	Number of cycles	Model of sequence evolution
DMRT1	Doublesex and Mab-3 related Transcription Factor 1	1	Exon 2	180 bp	F: 5' TGAGAAGGCAGCAGGCTC 3'<br>R: 5' CTGTAGAGCTGGTCGATGCTAGT 3'	0	0.05	50°C	35	GTR + I
P450	Aromatase	3	2 Exons + 1 Intron	320 bp	F: 5' AAAGATTCAGAAATACTTTGACG 3'<br>R: 5' GCAAAAATAAGCTCTGTTGC 3'	0	0.05	50°C	35	TN93 + I
SF-1	Steroidogenic Factor 1	19	Exon 3	595-598 bp	F: 5' CTGTTCGTGCTGACCGAAT 3'<br>R: 5' GAGTCTGATCGGCCATCTTG 3'	0.4	0.05	55°C	38	GTR + Γ + I
SPAG6	Sperm associated Antigene 6	6	Exon 5	187 bp	F: 5' GTCACAAGCTGTGGTGGATG 3'<br>R: 5' CTGGGGAGAGAATCATCTGG 3'	0.4	0.05	55°C	38	K80 + I
SOX3	SRY (sex determining region Y)-box 3	8	Exon 1	779-785 bp	F: 5' GCATGYTGGACACAGACATC 3'<br>R: 5' GCTGATCATATCTCGCAGGTC 3'	0	0.05	50°C	35	GTR + Γ + I
VLDLR	Vitellogenine Receptor or Very Low Density Lipoprotein Receptor	1	Intron	651-693 bp	F: 5' GACCAAGTGGCATTGTGTTG 3'<br>R: 5' CGATCATTAATCTGTGGAGCA 3'	0.6	0.1	54=>48°C	40	GTR + Γ + I

Table S2: Marker name and information about the PCR protocols and the specific models of sequence evolution used for trees in Figs. S2 to S7.

Details on the molecular dating approach for nuclear and mitochondrial data

Molecular dating was performed in BEAST v. 1.8.3 [12] (with input files created in BEAUTi v. 1.8.3). For the nuclear data set, we optimized the partitioning scheme by initially treating each gene fragment separately and determined the most suitable substitution models using PartitionFinder v.1.1.1 [13]. We evaluated only models available in BEAST with the following settings: branch lengths linked, Bayesian Information Criterion (BIC) for model selection, and a greedy search algorithm. Divergence time analyses were then run using partitions and models as selected by PartitionFinder, resulting in two partitions (p1: comprising genes *dmrt1, p450, sf1*, *sox3*, *spag6*: model K80+I+G; and p2, comprising *vldlr*: model TrN+I+G), with substitution schemes unlinked between partitions. We included *Bufo bufo* as outgroup to allow for calibration on the root node, and imposed the following age constraints to the molecular clock: (1) A mean age of 1.80 My for the split between *B. boulengeri* and *B. siculus* with a normal distributed range (SD = 0.61; 95% HPDI: 3.00-0.60), based on their previously obtained divergence date [14]; (2) a mean age of 2.50 My for the divergence between lineages of *B. boulengeri* and *B. siculus* vs. the monophyletic lineage comprising *B. balearicus* and *B. viridis*, assuming a normal distribution (SD = 0.51; 95% HPDI: 3.55-1.53 My; [15]); (3) a minimum age of 18 My for the most recent common ancestor (MRCA) of *Bufo bufo* and the *B. viridis* subgroup, following the settings used by Garcia-Porta *et al.* [16] (2012; gamma distribution, shape = 1.2, scale = 4, offset = 18 My, 95% HPDI: 18.2-34.4 My) for this divergence. In the absence of appropriate fossils that can be related to specific mtDNA lineages [5], these are the best available calibration points for divergence estimations in Palearctic green toads [14].

For the mtDNA data (D-loop), we selected the best-fit model of evolution through jModelTest v2.1.7 based on BIC [17,18] (HKY + G), and followed the workflow as described above. Since *B. bufo* mtDNA could not aligned to green toad D-loops due to large INDELs, we included *B. raddei* as an outgroup. In addition to the age constraints (1) and (2) as implemented for the analyses of the nuclear data set (see above), we calibrated the ingroup (including stem) to have a minimum age of 18 My based on the oldest known green toad fossils [19]; cf. [5]. We applied a lognormal prior age distribution on that calibration point with an upper range bound of 29.5 My (mean = 0.48, SD = 1, offset = 18 My, 95% HPDI: 18.2-29.5 My; [20]).

Text S3: Amplification success of the nuclear markers and sequence number per species

CYP19 – Amplification was successful in the 51 individuals with one single consensus sequence in *B. balearicus*, *B. boulengeri*, *B. latastii*, *B. shaartusiensis*, *B. siculus*, *B. turanensis*, *B. variabilis* and *B. viridis*, two sequences in *B. luristanicus* and *B. surdus*, three sequences in *B. baturae*, *B. zugmayeri* and *B. pseudoraddei*, between three to four sequences of *B. oblongus* and *B. pewzowi* (Fig. S3).

DMRT1 – Amplification was successful in the 51 individuals with one single consensus sequence in *B. balearicus*, *B. boulengeri*, *B. luristanicus*, *B. shaartusiensis*, *B. siculus*, *B. surdus*, *B. turanensis* and *B. viridis*, two sequences in *B. variabilis*, between one to two sequences for *B. latastii*, three sequences in *B. zugmayeri* and *B. pseudoraddei*, between two to three sequences of *B. baturae*, between two to four sequences of *B. pewzowi* and *B. oblongus* (Fig. S4).

SF-1 – Amplification was successful in the 51 individuals with one single consensus sequence in *B. balearicus*, *B. siculus*, *B. turanensis*, *B. variabilis* and *B. viridis*, two sequences in *B. boulengeri*, *B. luristanicus*, *B. pseudoraddei*, *B. surdus* and *B. zugmayeri*, , between one to two sequences for *B. latastii* and *B. shaartusiensis*, between two to three sequences in *B. baturae*, between two to four sequences of *B. oblongus* and *B. pewzowi* (Fig. S5).

SOX3 – No sequences successfully amplified for the individuals: L5, O3, PS1, T4 and Z1. We obtained one single consensus sequence in *B. balearicus*, *B. boulengeri*, *B. latastii*, *B. luristanicus*, *B. shaartusiensis*, *B. siculus*, *B. surdus*, *B. turanensis*, *B. variabilis* and *B. viridis*, between one to two sequences for *B. baturae* and *B. oblongus* and between one to two sequences for *B. baturae* (Fig. S6).

SPAG6 – Amplification was successful in the 51 individuals with one single consensus sequence in *B. balearicus*, *B. boulengeri*, *B. latastii*, *B. luristanicus*, *B. shaartusiensis*, *B. siculus*, *B. surdus*, *B. turanensis*, *B. variabilis* and *B. viridis*, two sequences in *baturae*, *B. oblongus, B. pseudoraddei, B. pewzowi* and *B. zugmayeri* (Fig. S7).

VLDLR – No sequences successfully amplified for the individuals of *B. shaartusiensis* and the individuals O3 and O5. We obtained one single consensus sequence in *B. boulengeri*, *B. luristanicus*, *B. siculus*, *B. turanensis*, and *B. viridis*, two sequences of *B. surdus* and *B. variabilis*, three sequences of *B. pseudoraddei* and *B. zugmayeri*, between one to two sequences for *B. balearicus*, *baturae* and *B. latastii*, between one to two sequences of *B. oblongus* and *B. pewzowi* (Fig. S8).

In several markers, some allotetraploid individuals exhibited three alleles clustering with one parental lineage (i.e. maternal or paternal) whereas the remaining allele clustered with the other one (i.e. paternal or maternal respectively) or displayed only alleles from one paternal cluster. In *DMRT1* the individual P4 displayed three paternal alleles and only one maternal allele whereas in *P450* only three paternal copies and no maternal copy were detected in the individuals O4 and P1. In *SOX3*, only two paternal alleles were detected for O5 and O1, and one paternal copy for P3 and P5.

Amplification of nuclear markers for three initially unidentified individuals

Thirteen out of the 30 microsatellite markers, developed by Betto-Colliard *et al*. [21], were used to infer the ploidy level of the four unidentified individuals, using the same PCR protocols (*Ba*C101, *Ba*C123, *Ba*C201, *Ba*D5, *Bl*B118, *Bl*B3, *Bl*B7, *Bl*D102, *Bl*D114, *Bl*D115, *Bl*D118, *Bl*D140, *Bl*D214). The unidentified individual X1 exhibited four different alleles in three markers (*Bl*B7, *Bl*D102, *Bl*D115). The unidentified individual X3 showed four different alleles in seven markers (*Bl*B118, *Bl*B3, *Bl*B7, *Bl*D114, *Bl*D118, *Bl*D140, *Bl*D214). The unidentified individual X4 displayed a maximum of three different alleles in seven markers (*Ba*C101, *Bl*B118, *Bl*B7, *Bl*D102, *Bl*D115, *Bl*D118, *Bl*D140).

Supplementary Text S4: Maximum likelihood tests on the placement of *B. turanensis* in the maternal clade of the Bayesian tree, shown in Fig. 1c

We tested the resulting placement of the *turanensis* clade in the best BI tree (Fig. 1c) against alternative topologies in a ML framework to evaluate whether likelihoods were significantly different.

Based on the mtDNA we performed the approximately unbiased (AU) [23] and Shimodaira-Hasegawa (SH) [24] tests between the following relationships:

t1 = best BI, (((2n *turanensis*),4n *pewzowi*),(4n *oblongus*),2n *variabilis*, 2n *balearicus*, 2n viridis);

t2 = (((2n *turanensis*,4n *pewzowi*),4n *oblongus*),2n *variabilis*, 2n *balearicus*, 2n *viridis*);

t3 = ((2n *turanensis*,(4n *pewzowi*, 4n *oblongus*)), 2n *variabilis*, 2n *balearicus*, 2n *viridis*);

t4 = ((4n *pewzowi*, 4n *oblongus*),(2n *turanensis*, 2n *balearicus*, 2n *viridis*)).

We estimated the four phylogenies as above and the per-site likelihoods in RAxML 8.2.7 [25]. The p-values were then obtained using the program CONSEL [26].

Although the p-value was the highest for model 1 (best BI topology: *B. turanensis* monophyletic, *B. pewzowi* basally) in both, the AU and SH tests, none of the topologies were rejected (Table S3).

Table S3: Summarized results of the topological tests of different phylogenetic relationships (t1-t4) of *B. turanensis* for the mitochondrial data set. Shown are the p-values of the proximately unbiased (au) test, the Shimodaira–Hasegawa (sh) test and the weighted SH (wsh) test. Best-supported topology is set in **bold**.

Topology (t)	au	sh	wsh	df
**t1: best BI, (((2n *turanensis*),4n *pewzowi*),(4n *oblongus*),2n *variabilis*, 2n *balearicus*, 2n *viridis*)**	**0.701**	**0.793**	**0.806**	**8**
t2: (((2n *turanensis*,4n *pewzowi*),4n *oblongus*),2n *variabilis*, 2n *balearicus*, 2n *viridis*)	0.572	0.733	0.747	8
t3: ((2n *turanensis*,(4n *pewzowi*, 4n *oblongus*)), 2n *variabilis*, 2n *balearicus*, 2n *viridis*)	0.196	0.312	0.267	8
t4: ((4n *pewzowi*, 4n *oblongus*),(2n *turanensis*, 2n *balearicus*, 2n *viridis*))	0.178	0.320	0.314	8

Suppl. Text S5: Details on the five allopolyploidization events (Fig. S2: I-V)

Details on the five allopolyploidization events (Fig.S2: I-V) that resulted in taxonomically recognized species and signatures of three additional genomic interactions that led to allopolyploids (Fig. S2: UIL X1, X2, X4)

I: 4n allotetraploid (*Bufo pewzowi*). Within the western clade (Fig. 1c), the subclade of allotetraploid *B. pewzowi* contains a single diploid species, *B. turanensis*, that also occurs geographically proximate, suggesting its lineage is the most parsimonious candidate maternal genome donor. The paternal genome is probably inherited from a species that had earlier diverged from *B. latastii* (Fig. 1c). Maternal (0.82 My; 0.32-1.45 My) and paternal (1.45, 0.54-2.64 My) subclades of 4n *Bufo pewzowi* are both estimated to have started their phylogenetic diversification in Pleistocene periods, suggesting this as a timeframe for the allopolyploidization event. In the mtDNA- and maternal sub-genome trees, *B. pewzowi* forms a paraphyletic group, which may indicate several diverged subpopulations or, alternatively, multiple origins of the allopolyploid *B. pewzowi* (Fig. 1b, c). A surprising result in this context is the phylogenetic position of 2n *B. turanensis*, which looks as if the recent representatives of this diploid lineage have been derived from the tetraploid *B. pewzowi*, both in the nuclear and the mtDNA phylogenies (i.e. after allopolyploidization in the Pleistocene, the true maternal *B. turanensis* ancestral lineage got extinct but later re-emerged by loss of the paternal genome from the 4n *B. pewzowi*). To test whether this is a true evolutionary signature or rather a phylogenetic artifact, we performed maximum likelihood ratio tests on the maternal nuclear ancestry (Suppl. Text S4). The data at hand indeed provide the highest likelihood for the topology suggesting that *B. turanensis* is derived from 4n

B. pewzowi. However, alternative topologies could not be rejected and a final clarification of this question exceeds the framework of our study. However, future work should indeed test the intriguing possibility that a diploid vertebrate taxon could have evolved from an allotetraploid ancestor by the loss of a genome.

II: 4n *B. oblongus*. The situation is less clear for 4n *B. oblongus* from northern and eastern Iran, whose maternal genome forms a sister clade to the maternal ancestor of *B. pewzowi*, from which *B. oblongus* is cytogenetically distinguished [8]. Tetraploid *B. oblongus* presumably also inherited its maternal subgenome from the lineage represented by diploid *B. turanensis*, while its paternal subgenome is derived from a lineage related to *B. latastii* (Fig. 1c). Within the paternal clade of *B. oblongus*, we find at least one inconsistency (O5), which either indicates that this tetraploid arose several times (Fig. 1c), or experienced recent genetic interactions with diploid or triploid forms, as documented from Iran [27]. The maternal clade comprising 2n *B. turanensis,* 4n *B. oblongus* and 4n *B. pewzowi*, forms a sister taxon to the European diploids (*B. variabilis, B. viridis* and *B. balearicus*), with a closer relationship with geographically proximate *B. variabilis* suggested by the mtDNA tree.

III: 3n *B. baturae.* The maternal ancestor that provided the haploid clonal genome of 3n *B. baturae* is a mid-Pleistocene (1.3 Mya, 0.64-2.24 Mya, Fig. 1c) derivation from diploid *B. shaartusiensis*, a relationship which is also well-supported by the mtDNA phylogeny (Fig. 1b). The paternal ancestor is a lower Pleistocene (2.0 My) sister taxon of the paternal genomes of allotriploid *B. zugmayeri* and *B. pseudoraddei*, which are together broadly related to diploid *B. latastii* (Figs. 1c).

IV: 3n *B. zugmayeri.* According to the mtDNA-phylogeny (Fig. 1b), the maternal ancestor of allotriploid *B. zugmayeri* stems from the same clade as 2n *B. turanensis,* 4n *B. oblongus* and 4n *B. pewzowi*, whereas its nuclear maternal ancestry rather points to a relationship with 2n *B. shaartusiensis* as well as a strongly supported Pleistocene (0.93 My, 0.22-2.1 My) sister relationship with the maternal genome of 3n *B. pseudoraddei* (Fig. 1c, Fig. S1). This suggests a more complex allopolyploid formation than for both *B. baturae* and *B. pseudoraddei*. The paternal ancestry of *B. zugmayeri* shows a Pleistocene sister-relationship to the paternal subgenome of *B. pseudoraddei*. Both of these are closely related to the paternal ancestor of *B. baturae* and some Iranian tetraploid forms (UIL X1, X2), and all of which represent diverged lineages derived from ancestors of *B. latastii* (Fig. 1c).

V: 3n *B. pseudoraddei.* While the nuclear ancestry of *B. pseudoraddei*, endemic to the Swat valley of Pakistan, appears at first glance similar to that of 3n *B. zugmayeri*, quantitatively different genome contributions led to its formation. Namely, *B. pseudoraddei* presents 2

sets of maternal subgenomes, *versus* only one for *B. zugmayeri* (Fig. 1c, Fig. S1). In addition, *B. pseudoraddei*'s mtDNA is closely related to that of *B. shaartusiensis* (and 3n *B. baturae*), while that of *B. zugmayeri* is closer to *B. turanensis* (Fig. 1b).

Signatures of three additional genomic interactions that led to allopolyploids (Fig. S2: UIL X1, X2, X4)

While on the one hand, UIL X1 and X2 from northeastern Iran share their mtDNA (Fig. 1b) and maternal nuDNA phylogenetic history with *B. oblongus* (of which they might simply represent a sub-population), their paternal genomes present the lineage of the paternal ancestry of 3n *B. baturae*. As mentioned for O5 (see above), this either indicates that *B. oblongus*-like tetraploids arose several times (Fig. 1c), or reflects recent genetic interactions with diploid or triploid forms in Iran [27]. Triploid UIL X4, on the other hand, relates to 2n *B. shaartusiensis* both in terms of mtDNA haplotype (Fig. 1b) and nuclear maternal genome (Fig. 1c), while its paternal genome clusters with the paternal ancestry of recent 4n *B. pewzowi*, suggesting this is a recent hybrid between a *B. shaartusiensis* mother and a *B. pewzowi* father, found at the type locality of the maternal species. It is worth noting that UIL X4 displayed two maternal alleles for *SOX3* (Fig. S5): one related to *B. baturae* and another one to *B. pewzowi* maternal lineage (which is not apparent in Fig. 1c because our concatenation approach only kept one maternal sequence).

References:

1. Dufresnes C, Bonato L, Novarini N, *et al.* 2014 Inferring the degree of incipient speciation in secondary contact zones of closely related lineages of Palearctic green toads *Bufo viridis* subgroup. *Hered.* **113**, 9-20 (doi:10.1038/hdy.2014.26).
2. Colliard C, Sicilia A, Turrisi GF, *et al.* 2010: Strong reproductive barriers in a narrow hybrid zone of West-Mediterranean green toads *Bufo viridis* subgroup with Plio-Pleistocene divergence. *BMC Evol. Biol.* **10**, 232 (doi:10.1111/j.1755-0998.2009.02600.x).
3. Stöck M, Ustinova J, Lamatsch DK, *et al.* 2010. A vertebrate reproductive system involving three ploidy levels: hybrid origin of triploids in a contact zone of diploid and tetraploid Paleartic green toads *Bufo viridis* subgroup. *Evol.* **64,** 944-959 (doi:10.1111/j.1558-5646.2009.00876.x).
4. Betto-Colliard C, Sermier R, Litvinchuk S, *et al.* 2015 Origin and genome evolution of polyploid green toads in Central Asia: evidence from microsatellite markers. *Hered.* **114**, 300-308 (doi:10.1038/hdy.2014.100).

5. Stöck M, Moritz C, Hickerson M, *et al.* 2006. Evolution of mitochondrial relationships and biogeography of Palearctic green toads *Bufo viridis* subgroup with insights in their genomic plasticity. *Mol. Phylogen. Evol.* **41,** 663-689 (doi:10.1016/j.ympev.2006.05.026).
6. Litvinchuk SN, Mazepa GO, Pasynkova RA, *et al.* 2011 Influence of environmental conditions on the distribution of Central Asian green toads with three ploidy levels. *J. Zool. Syst. Evol. Res.* **9,** 233-239 (doi:10.1111/j.1439-0469.2010.00612.x).
7. Bogart JP 2003 Genetics and systematics of hybrid species. In *Reproductive Biology and Phylogeny of Urodela* (ed DM Sever), pp. 109–134. Enfield, New Hampshire: Science Publishers Inc.
8. Stöck M, Steinlein C, Lamatsch DK, *et al.* 2005 Multiple origins of tetraploid taxa in the Eurasian *Bufo viridis* subgroup. *Genetica*, **124**, 255–272 (doi:10.1007/s10709-005-3085-9).
9. Stöck M, Ustinova J, Betto-Colliard C, *et al.* 2012 Simultaneous Mendelian and clonal genome transmission in a sexually reproducing all-triploid vertebrate. *Proc. R. Soc. B* **279,** 1293-1299 (doi:10.1098/rspb.2011.1738).
10. Broquet T, Berset-Braendli L, Emaresi G, *et al.* 2005 Buccal swabs allow efficient and reliable microsatellite genotyping in amphibians. *Conserv. Genet.* **8**, 509–511 (doi: 10.1007/s10592-006-9180-3).
11. Guindon S, Dufayard JF, Lefort V, *et al.* 2010 New algorithms and methods to estimate Maximum-Likelihood phylogenies: assessing the performance of PhyML 3.0. *Syst. Biol.* **59**, 307-321 (doi:10.1093/sysbio/syq010).
12. Drummond AJ, Rambaut, A 2007 BEAST: Bayesian evolutionary analysis by sampling trees. *BMC Evol. Biol.* **7,** 214 (doi:10.1093/sysbio/syq010).
13. Lanfear, R, Calcott, B, Ho SY, Guindon, S (2012) Partitionfinder: combined selection of partitioning schemes and substitution models for phylogenetic analyses. *Mol. Biol. Evol.* **29**, 1695-1701 (doi.org/10.1093/molbev/mss020).
14. Stöck M, Sicilia A, Belfiore N, *et al.* (2008). Post-Messinian evolutionary relationships across the Sicilian channel: Mitochondrial and nuclear markers link a new green toad from Sicily to African relatives. *BMC Evol. Biol.* **8**, 56 (doi.org/10.1186/1471-2148-8-56).
15. Dufresnes C, Bonato L, Novarini N, *et al.* 2014 Inferring the degree of incipient speciation in secondary contact zones of closely related lineages of Palearctic green toads *Bufo viridis* subgroup. *Hered.* **113**, 9-20 (doi:10.1038/hdy.2014.26).

16. Garcia-Porta J, Litvinchuk SN, Crochet PA, *et al.* (2012) Molecular phylogenetics and historical biogeography of the west-Palearctic common toads (*Bufo bufo* species complex). *Mol. Phylogen. Evol.* **63**, 113–130 (doi.org/10.1016/j.ympev.2011.12.019).
17. Posada D. 2008. jModelTest: Phylogenetic Model Averaging. *Mol. Biol. Evol.* **25**, 1253-1256 (doi.org/10.1093/molbev/msn083).
18. Darriba D, Taboada GL, Doallo R, Posada D 2012 jModelTest 2: more models, new heuristics and parallel computing. *Nat. Meth*. **9**, 772 (doi:10.1038/nmeth.2109).
19. Böhme, M, 2003 The Miocene climatic optimum: evidence from ectothermic vertebrates of Central Europe. *Palaeogeo. Palaeoclimat. Palaeoecolog.* **195**, 389–401 (doi.org/10.1016/S0031-0182(03)00367-5).
20. Portik D.M. Papenfuss T.J. (2015) Historical biogeography resolves the origins of endemic Arabian toad lineages (Anura: Bufonidae): Evidence for ancient vicariance and dispersal events with the Horn of Africa and South Asia. *BMC Evol. Biol.* **15**, 152 (DOI 10.1186/s12862-015-0417-y).
21. Betto-Colliard C., Sermier R., Perrin N. & Stöck M. (2013): Development and cross-amplification of thirty microsatellite loci in five diploid and polyploid Central Asian species of Palearctic green toads (*Bufo viridis* subgroup). Conservation Genetics Resources 5: 242-249 (: 10.1007/s12686-012-9779-2).
22. Shimodaira H 2002 An approximately unbiased test of phylogenetic tree selection. *Syst. Biol.* **51,** 492-508 (doi.org/10.1080/10635150290069913).
23. Shimodaira H, Hasegawa M 1999 Multiple comparisons of log-likelihoods with applications to phylogenetic inference. *Mol. Biol. Evol.* **16,** 1114-1116 (ISSN: 0737-4038).
25. Stamatakis A 2014 RAxML Version 8: A tool for phylogenetic analysis and post-analysis of large phylogenies. *Bioinf.* **30,** 1312-1313 (doi.org/10.1093/ bioinformatics/btu033).
26. Shimodaira H Hasegawa M 2001 CONSEL: for assessing the confidence of phylogenetic tree selection. *Bioinf.* **17,** 1246-1247 (doi.org/10.1093/ bioinformatics/17.12.1246).
27. Fakharzadeh F, Darvish J, Kami HG, *et al.* 2015 Discovery of triploidy in Palearctic green toads Anura: Bufonidae from Iran with indications for a reproductive system involving diploids and triploids. *Zool. Anz. J. Comp. Zool.* **255**, 25–31.

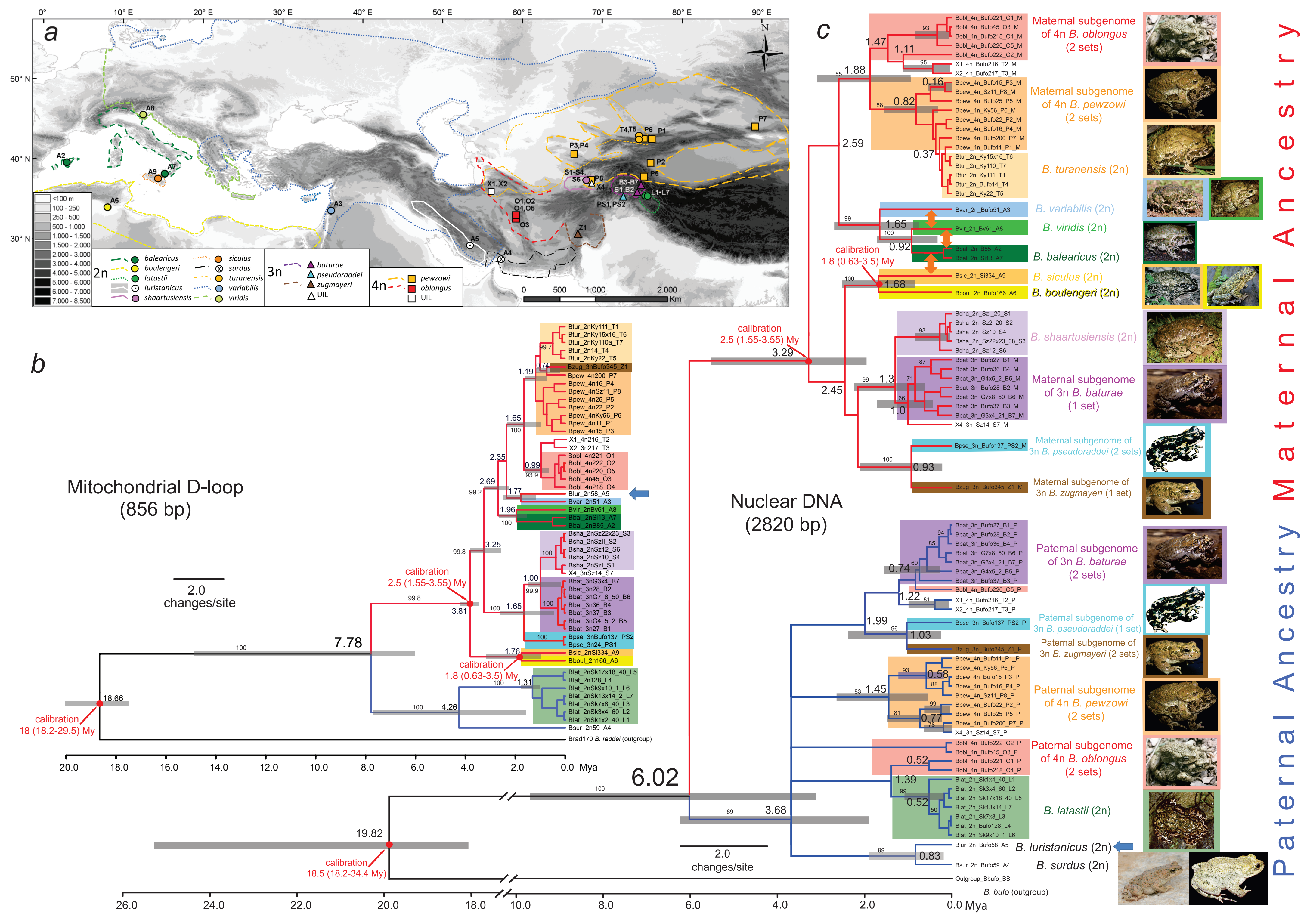

a
b
c
2n
3n
4n
balearicus
boulengeri
latastii
luristanicus
shaartusiensis
siculus
surdus
turanensis
variabilis
viridis
baturae
pseudoraddei
zugmayeri
UIL
pewzowi
oblongus
Mitochondrial D-loop
(856 bp)
2.0
changes/site
calibration
2.5 (1.55-3.55) My
calibration
1.8 (0.63-3.5) My
calibration
18 (18.2-29.5) My
Brad170 B. raddei (outgroup)
Nuclear DNA
(2820 bp)
calibration
18.5 (18.2-34.4 My)
Maternal subgenome of 4n B. oblongus (2 sets)
Maternal subgenome of 4n B. pewzowi (2 sets)
B. turanensis (2n)
B. variabilis (2n)
B. viridis (2n)
B. balearicus (2n)
B. siculus (2n)
B. boulengeri (2n)
B. shaartusiensis (2n)
Maternal subgenome of 3n B. baturae (1 set)
Maternal subgenome of 3n B. pseudoraddei (2 sets)
Maternal subgenome of 3n B. zugmayeri (1 set)
Paternal subgenome of 3n B. baturae (2 sets)
Paternal subgenome of 3n B. pseudoraddei (1 set)
Paternal subgenome of 3n B. zugmayeri (2 sets)
Paternal subgenome of 4n B. pewzowi (2 sets)
Paternal subgenome of 4n B. oblongus (2 sets)
B. latastii (2n)
B. luristanicus (2n)
B. surdus (2n)
B. bufo (outgroup)
Maternal Ancestry
Paternal Ancestry
Mya

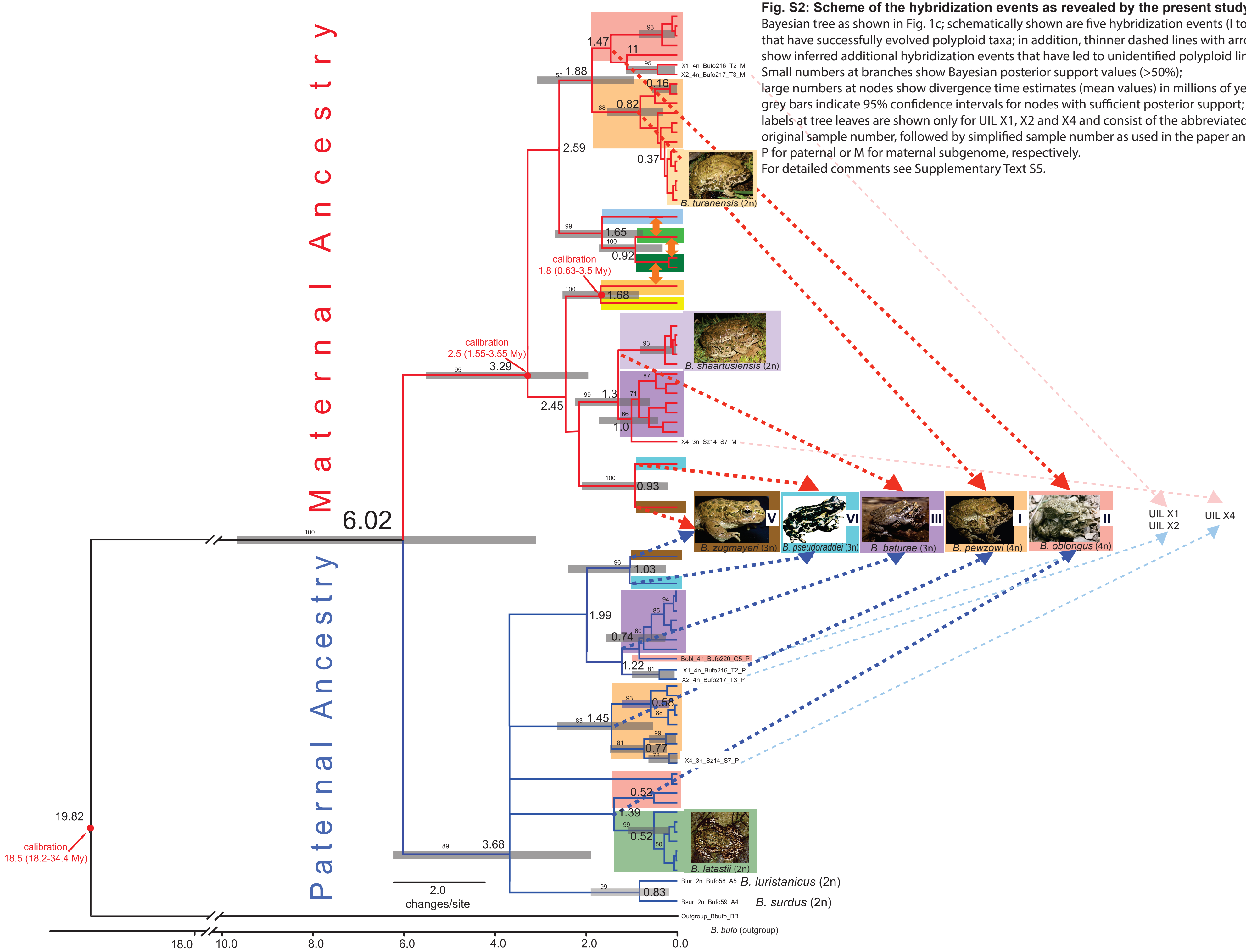

Fig. S2: Scheme of the hybridization events as revealed by the present study.
Bayesian tree as shown in Fig. 1c; schematically shown are five hybridization events (I to V; bold arrows) that have successfully evolved polyploid taxa; in addition, thinner dashed lines with arrows show inferred additional hybridization events that have led to unidentified polyploid lineages (UIL).
Small numbers at branches show Bayesian posterior support values (>50%);
large numbers at nodes show divergence time estimates (mean values) in millions of years ago (Mya);
grey bars indicate 95% confidence intervals for nodes with sufficient posterior support;
labels at tree leaves are shown only for UIL X1, X2 and X4 and consist of the abbreviated taxon name, original sample number, followed by simplified sample number as used in the paper and P for paternal or M for maternal subgenome, respectively.
For detailed comments see Supplementary Text S5.

Cyp-19

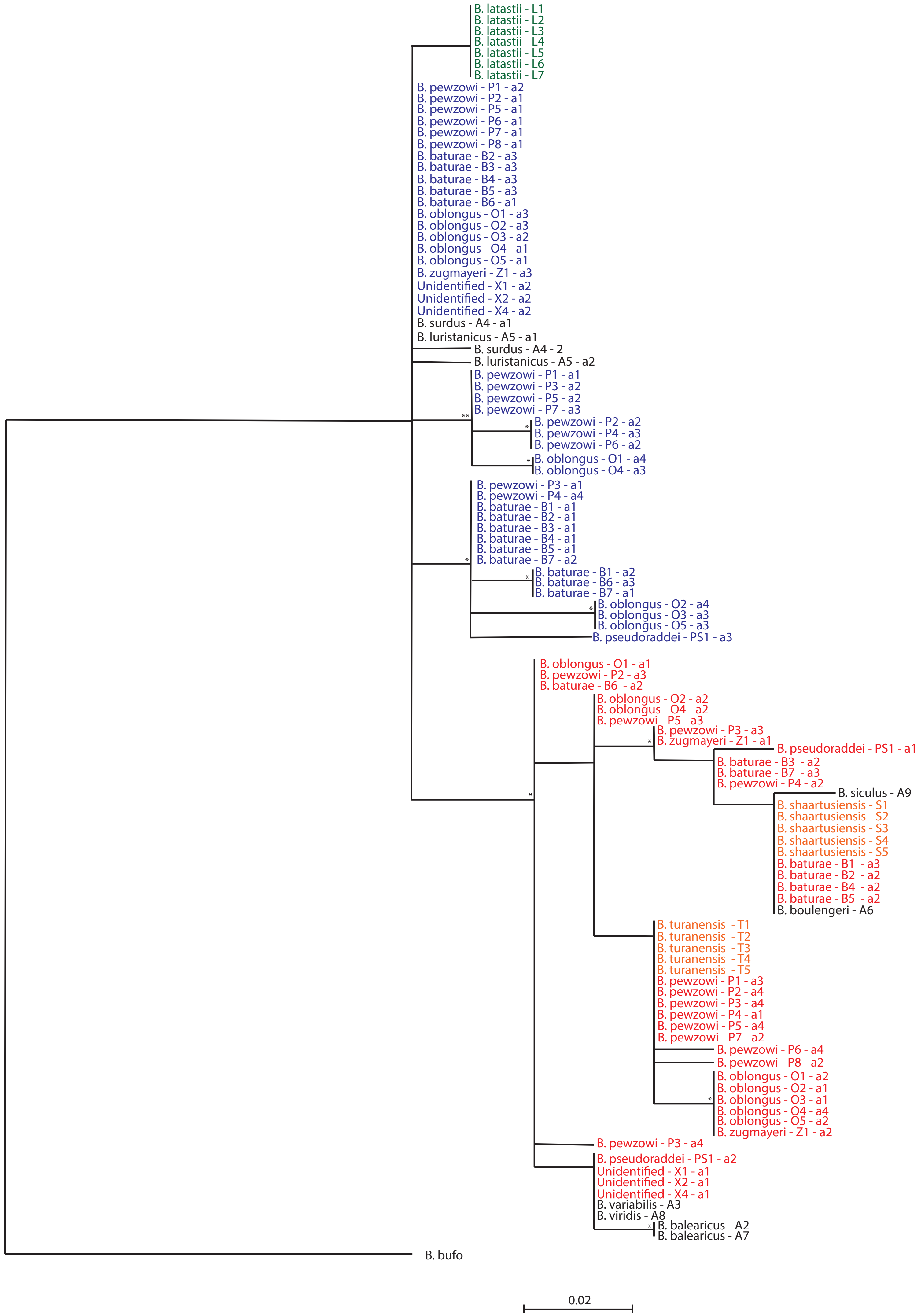
B. latastii - L1
B. latastii - L2
B. latastii - L3
B. latastii - L4
B. latastii - L5
B. latastii - L6
B. latastii - L7
B. pewzowi - P1 - a2
B. pewzowi - P2 - a1
B. pewzowi - P5 - a1
B. pewzowi - P6 - a1
B. pewzowi - P7 - a1
B. pewzowi - P8 - a1
B. baturae - B2 - a3
B. baturae - B3 - a3
B. baturae - B4 - a3
B. baturae - B5 - a3
B. baturae - B6 - a1
B. oblongus - O1 - a3
B. oblongus - O2 - a3
B. oblongus - O3 - a2
B. oblongus - O4 - a1
B. oblongus - O5 - a1
B. zugmayeri - Z1 - a3
Unidentified - X1 - a2
Unidentified - X2 - a2
Unidentified - X4 - a2
B. surdus - A4 - a1
B. luristanicus - A5 - a1
B. surdus - A4 - 2
B. luristanicus - A5 - a2
B. pewzowi - P1 - a1
B. pewzowi - P3 - a2
B. pewzowi - P5 - a2
B. pewzowi - P7 - a3
**
*
B. pewzowi - P2 - a2
B. pewzowi - P4 - a3
B. pewzowi - P6 - a2
*
B. oblongus - O1 - a4
B. oblongus - O4 - a3
B. pewzowi - P3 - a1
B. pewzowi - P4 - a4
B. baturae - B1 - a1
B. baturae - B2 - a1
B. baturae - B3 - a1
B. baturae - B4 - a1
B. baturae - B5 - a1
*
B. baturae - B7 - a2
*
B. baturae - B1 - a2
B. baturae - B6 - a3
B. baturae - B7 - a1
*
B. oblongus - O2 - a4
B. oblongus - O3 - a3
B. oblongus - O5 - a3
B. pseudoraddei - PS1 - a3
B. oblongus - O1 - a1
B. pewzowi - P2 - a3
B. baturae - B6 - a2
B. oblongus - O2 - a2
B. oblongus - O4 - a2
B. pewzowi - P5 - a3
B. pewzowi - P3 - a3
*
B. zugmayeri - Z1 - a1
B. pseudoraddei - PS1 - a1
B. baturae - B3 - a2
B. baturae - B7 - a3
B. pewzowi - P4 - a2
B. siculus - A9
*
B. shaartusiensis - S1
B. shaartusiensis - S2
B. shaartusiensis - S3
B. shaartusiensis - S4
B. shaartusiensis - S5
B. baturae - B1 - a3
B. baturae - B2 - a2
B. baturae - B4 - a2
B. baturae - B5 - a2
B. boulengeri - A6
B. turanensis - T1
B. turanensis - T2
B. turanensis - T3
B. turanensis - T4
B. turanensis - T5
B. pewzowi - P1 - a3
B. pewzowi - P2 - a4
B. pewzowi - P3 - a4
B. pewzowi - P4 - a1
B. pewzowi - P5 - a4
B. pewzowi - P7 - a2
B. pewzowi - P6 - a4
B. pewzowi - P8 - a2
B. oblongus - O1 - a2
B. oblongus - O2 - a1
*
B. oblongus - O3 - a1
B. oblongus - O4 - a4
B. oblongus - O5 - a2
B. zugmayeri - Z1 - a2
B. pewzowi - P3 - a4
B. pseudoraddei - PS1 - a2
Unidentified - X1 - a1
Unidentified - X2 - a1
Unidentified - X4 - a1
B. variabilis - A3
B. viridis - A8
*
B. balearicus - A2
B. balearicus - A7
B. bufo
0.02

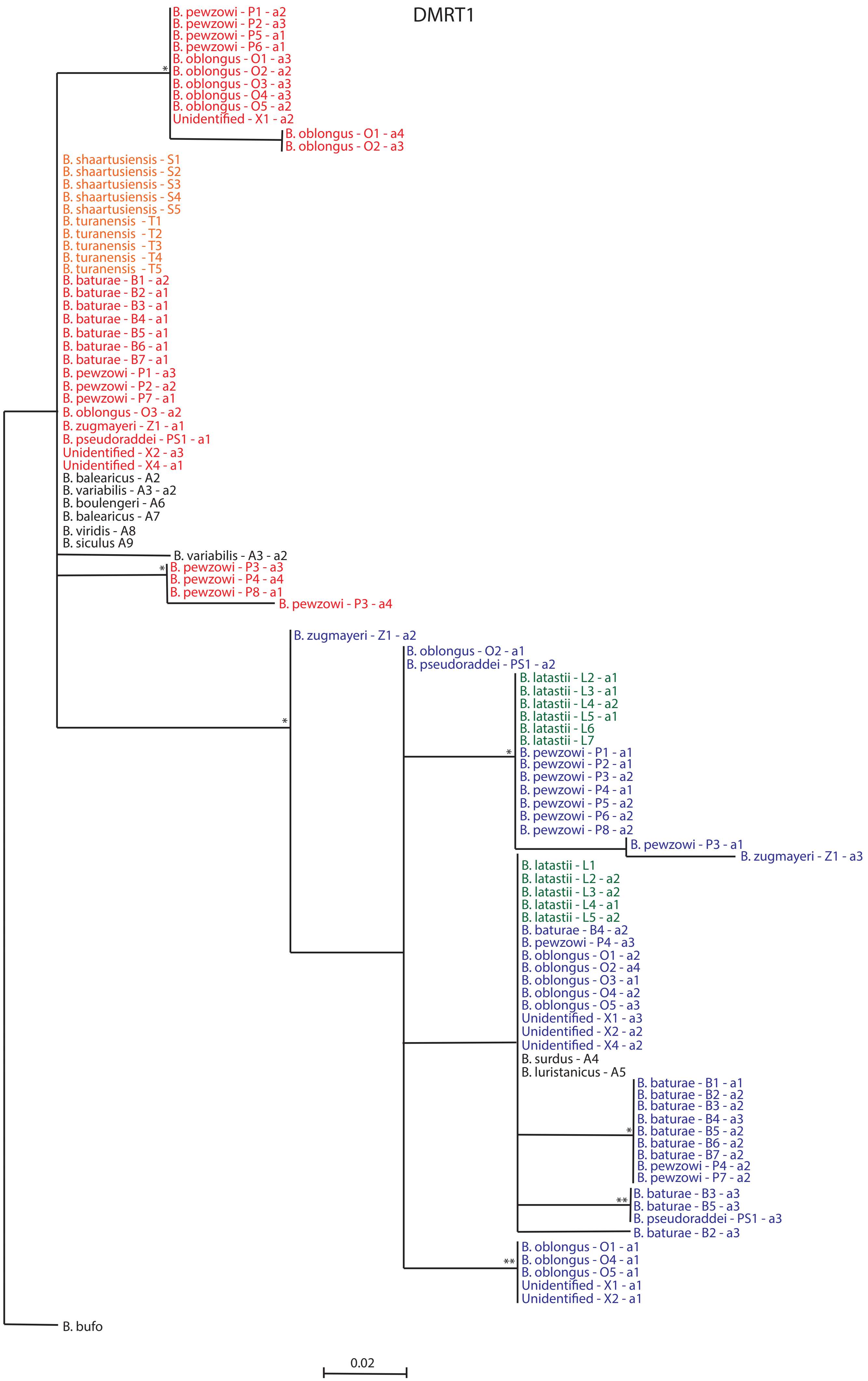
DMRT1
B. pewzowi - P1 - a2
B. pewzowi - P2 - a3
B. pewzowi - P5 - a1
B. pewzowi - P6 - a1
B. oblongus - O1 - a3
B. oblongus - O2 - a2
B. oblongus - O3 - a3
B. oblongus - O4 - a3
B. oblongus - O5 - a2
Unidentified - X1 - a2
B. oblongus - O1 - a4
B. oblongus - O2 - a3
B. shaartusiensis - S1
B. shaartusiensis - S2
B. shaartusiensis - S3
B. shaartusiensis - S4
B. shaartusiensis - S5
B. turanensis - T1
B. turanensis - T2
B. turanensis - T3
B. turanensis - T4
B. turanensis - T5
B. baturae - B1 - a2
B. baturae - B2 - a1
B. baturae - B3 - a1
B. baturae - B4 - a1
B. baturae - B5 - a1
B. baturae - B6 - a1
B. baturae - B7 - a1
B. pewzowi - P1 - a3
B. pewzowi - P2 - a2
B. pewzowi - P7 - a1
B. oblongus - O3 - a2
B. zugmayeri - Z1 - a1
B. pseudoraddei - PS1 - a1
Unidentified - X2 - a3
Unidentified - X4 - a1
B. balearicus - A2
B. variabilis - A3 - a2
B. boulengeri - A6
B. balearicus - A7
B. viridis - A8
B. siculus A9
B. variabilis - A3 - a2
B. pewzowi - P3 - a3
B. pewzowi - P4 - a4
B. pewzowi - P8 - a1
B. pewzowi - P3 - a4
B. zugmayeri - Z1 - a2
B. oblongus - O2 - a1
B. pseudoraddei - PS1 - a2
B. latastii - L2 - a1
B. latastii - L3 - a1
B. latastii - L4 - a2
B. latastii - L5 - a1
B. latastii - L6
B. latastii - L7
B. pewzowi - P1 - a1
B. pewzowi - P2 - a1
B. pewzowi - P3 - a2
B. pewzowi - P4 - a1
B. pewzowi - P5 - a2
B. pewzowi - P6 - a2
B. pewzowi - P8 - a2
B. pewzowi - P3 - a1
B. zugmayeri - Z1 - a3
B. latastii - L1
B. latastii - L2 - a2
B. latastii - L3 - a2
B. latastii - L4 - a1
B. latastii - L5 - a2
B. baturae - B4 - a2
B. pewzowi - P4 - a3
B. oblongus - O1 - a2
B. oblongus - O2 - a4
B. oblongus - O3 - a1
B. oblongus - O4 - a2
B. oblongus - O5 - a3
Unidentified - X1 - a3
Unidentified - X2 - a2
Unidentified - X4 - a2
B. surdus - A4
B. luristanicus - A5
B. baturae - B1 - a1
B. baturae - B2 - a2
B. baturae - B3 - a2
B. baturae - B4 - a3
B. baturae - B5 - a2
B. baturae - B6 - a2
B. baturae - B7 - a2
B. pewzowi - P4 - a2
B. pewzowi - P7 - a2
B. baturae - B3 - a3
B. baturae - B5 - a3
B. pseudoraddei - PS1 - a3
B. baturae - B2 - a3
B. oblongus - O1 - a1
B. oblongus - O4 - a1
B. oblongus - O5 - a1
Unidentified - X1 - a1
Unidentified - X2 - a1
B. bufo
0.02

SF-1

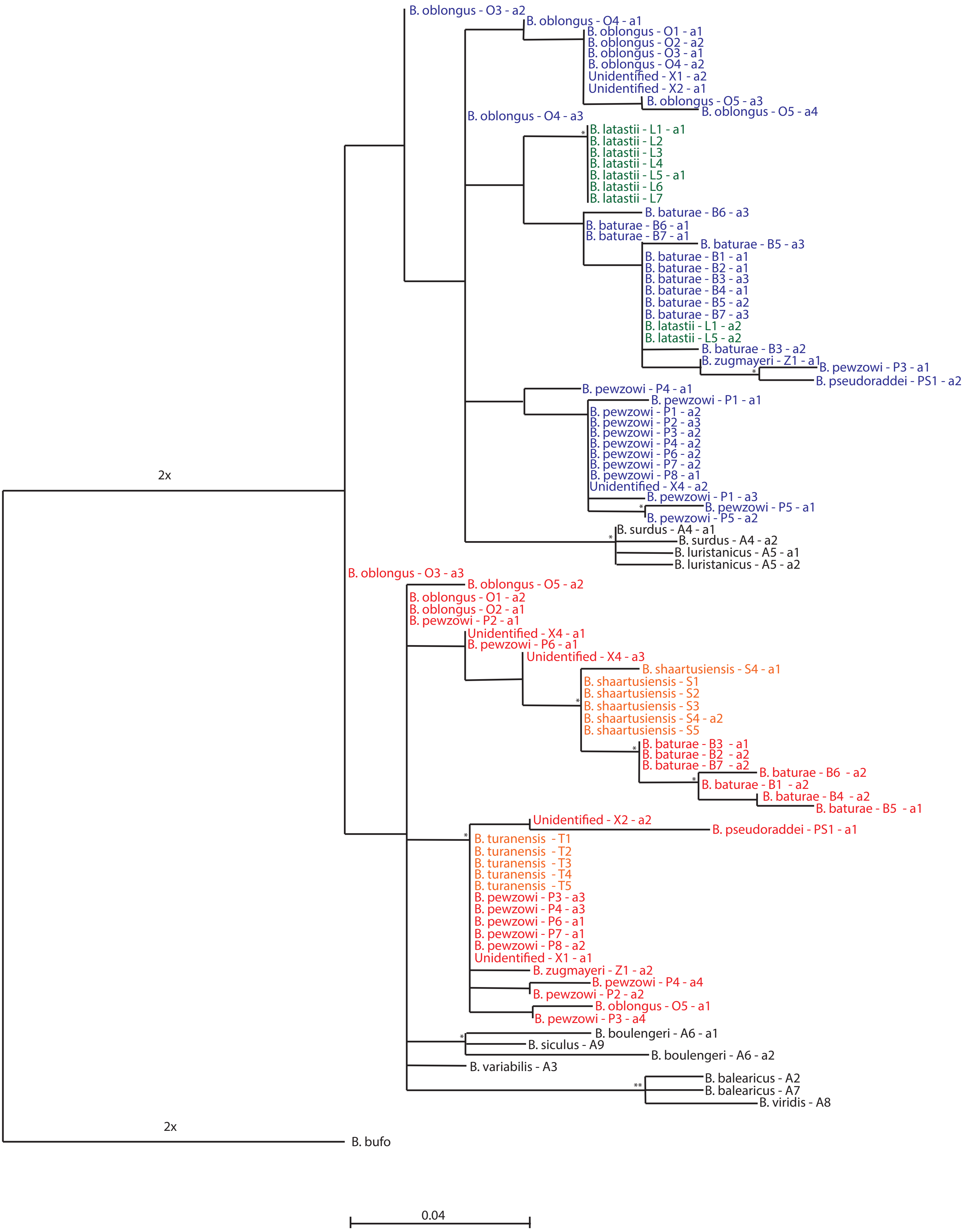
B. oblongus - O3 - a2
B. oblongus - O4 - a1
B. oblongus - O1 - a1
B. oblongus - O2 - a2
B. oblongus - O3 - a1
B. oblongus - O4 - a2
Unidentified - X1 - a2
Unidentified - X2 - a1
B. oblongus - O5 - a3
B. oblongus - O5 - a4
B. oblongus - O4 - a3
B. latastii - L1 - a1
B. latastii - L2
B. latastii - L3
B. latastii - L4
B. latastii - L5 - a1
B. latastii - L6
B. latastii - L7
B. baturae - B6 - a3
B. baturae - B6 - a1
B. baturae - B7 - a1
B. baturae - B5 - a3
B. baturae - B1 - a1
B. baturae - B2 - a1
B. baturae - B3 - a3
B. baturae - B4 - a1
B. baturae - B5 - a2
B. baturae - B7 - a3
B. latastii - L1 - a2
B. latastii - L5 - a2
B. baturae - B3 - a2
B. zugmayeri - Z1 - a1
B. pewzowi - P3 - a1
B. pseudoraddei - PS1 - a2
B. pewzowi - P4 - a1
B. pewzowi - P1 - a1
B. pewzowi - P1 - a2
B. pewzowi - P2 - a3
B. pewzowi - P3 - a2
B. pewzowi - P4 - a2
B. pewzowi - P6 - a2
B. pewzowi - P7 - a2
B. pewzowi - P8 - a1
Unidentified - X4 - a2
B. pewzowi - P1 - a3
B. pewzowi - P5 - a1
B. pewzowi - P5 - a2
B. surdus - A4 - a1
B. surdus - A4 - a2
B. luristanicus - A5 - a1
B. luristanicus - A5 - a2
2x
B. oblongus - O3 - a3
B. oblongus - O5 - a2
B. oblongus - O1 - a2
B. oblongus - O2 - a1
B. pewzowi - P2 - a1
Unidentified - X4 - a1
B. pewzowi - P6 - a1
Unidentified - X4 - a3
B. shaartusiensis - S4 - a1
B. shaartusiensis - S1
B. shaartusiensis - S2
B. shaartusiensis - S3
B. shaartusiensis - S4 - a2
B. shaartusiensis - S5
B. baturae - B3 - a1
B. baturae - B2 - a2
B. baturae - B7 - a2
B. baturae - B6 - a2
B. baturae - B1 - a2
B. baturae - B4 - a2
B. baturae - B5 - a1
Unidentified - X2 - a2
B. pseudoraddei - PS1 - a1
B. turanensis - T1
B. turanensis - T2
B. turanensis - T3
B. turanensis - T4
B. turanensis - T5
B. pewzowi - P3 - a3
B. pewzowi - P4 - a3
B. pewzowi - P6 - a1
B. pewzowi - P7 - a1
B. pewzowi - P8 - a2
Unidentified - X1 - a1
B. zugmayeri - Z1 - a2
B. pewzowi - P4 - a4
B. pewzowi - P2 - a2
B. oblongus - O5 - a1
B. pewzowi - P3 - a4
B. boulengeri - A6 - a1
B. siculus - A9
B. boulengeri - A6 - a2
B. variabilis - A3
B. balearicus - A2
B. balearicus - A7
B. viridis - A8
2x
B. bufo
0.04

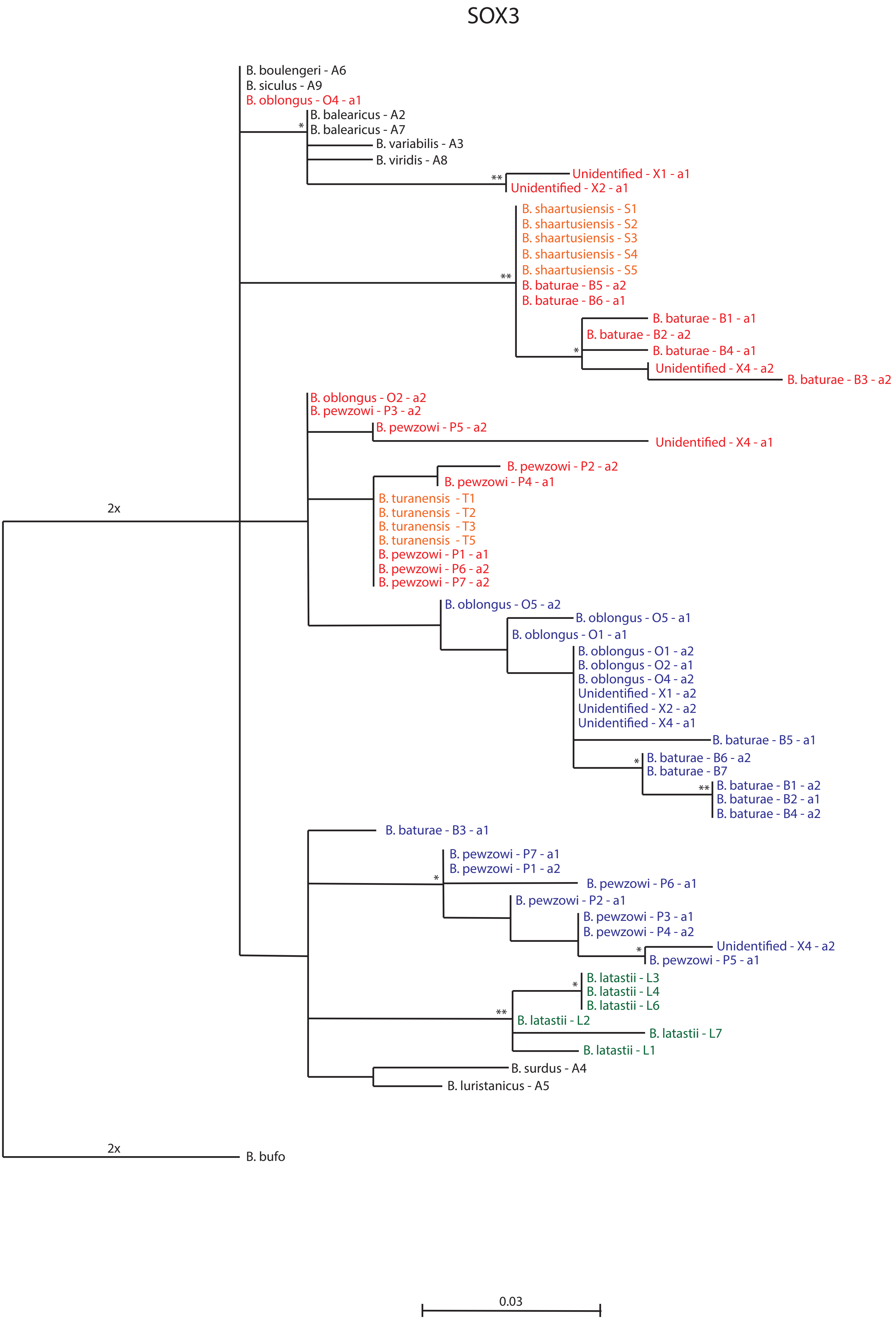
SOX3
B. boulengeri - A6
B. siculus - A9
B. oblongus - O4 - a1
*
B. balearicus - A2
B. balearicus - A7
B. variabilis - A3
B. viridis - A8
**
Unidentified - X1 - a1
Unidentified - X2 - a1
**
B. shaartusiensis - S1
B. shaartusiensis - S2
B. shaartusiensis - S3
B. shaartusiensis - S4
B. shaartusiensis - S5
B. baturae - B5 - a2
B. baturae - B6 - a1
*
B. baturae - B1 - a1
B. baturae - B2 - a2
B. baturae - B4 - a1
Unidentified - X4 - a2
B. baturae - B3 - a2
B. oblongus - O2 - a2
B. pewzowi - P3 - a2
B. pewzowi - P5 - a2
Unidentified - X4 - a1
B. pewzowi - P2 - a2
B. pewzowi - P4 - a1
2x
B. turanensis - T1
B. turanensis - T2
B. turanensis - T3
B. turanensis - T5
B. pewzowi - P1 - a1
B. pewzowi - P6 - a2
B. pewzowi - P7 - a2
B. oblongus - O5 - a2
B. oblongus - O5 - a1
B. oblongus - O1 - a1
B. oblongus - O1 - a2
B. oblongus - O2 - a1
B. oblongus - O4 - a2
Unidentified - X1 - a2
Unidentified - X2 - a2
Unidentified - X4 - a1
B. baturae - B5 - a1
*
B. baturae - B6 - a2
B. baturae - B7
**
B. baturae - B1 - a2
B. baturae - B2 - a1
B. baturae - B4 - a2
B. baturae - B3 - a1
B. pewzowi - P7 - a1
B. pewzowi - P1 - a2
*
B. pewzowi - P6 - a1
B. pewzowi - P2 - a1
B. pewzowi - P3 - a1
B. pewzowi - P4 - a2
*
Unidentified - X4 - a2
B. pewzowi - P5 - a1
*
B. latastii - L3
B. latastii - L4
B. latastii - L6
**
B. latastii - L2
B. latastii - L7
B. latastii - L1
B. surdus - A4
B. luristanicus - A5
2x
B. bufo
0.03

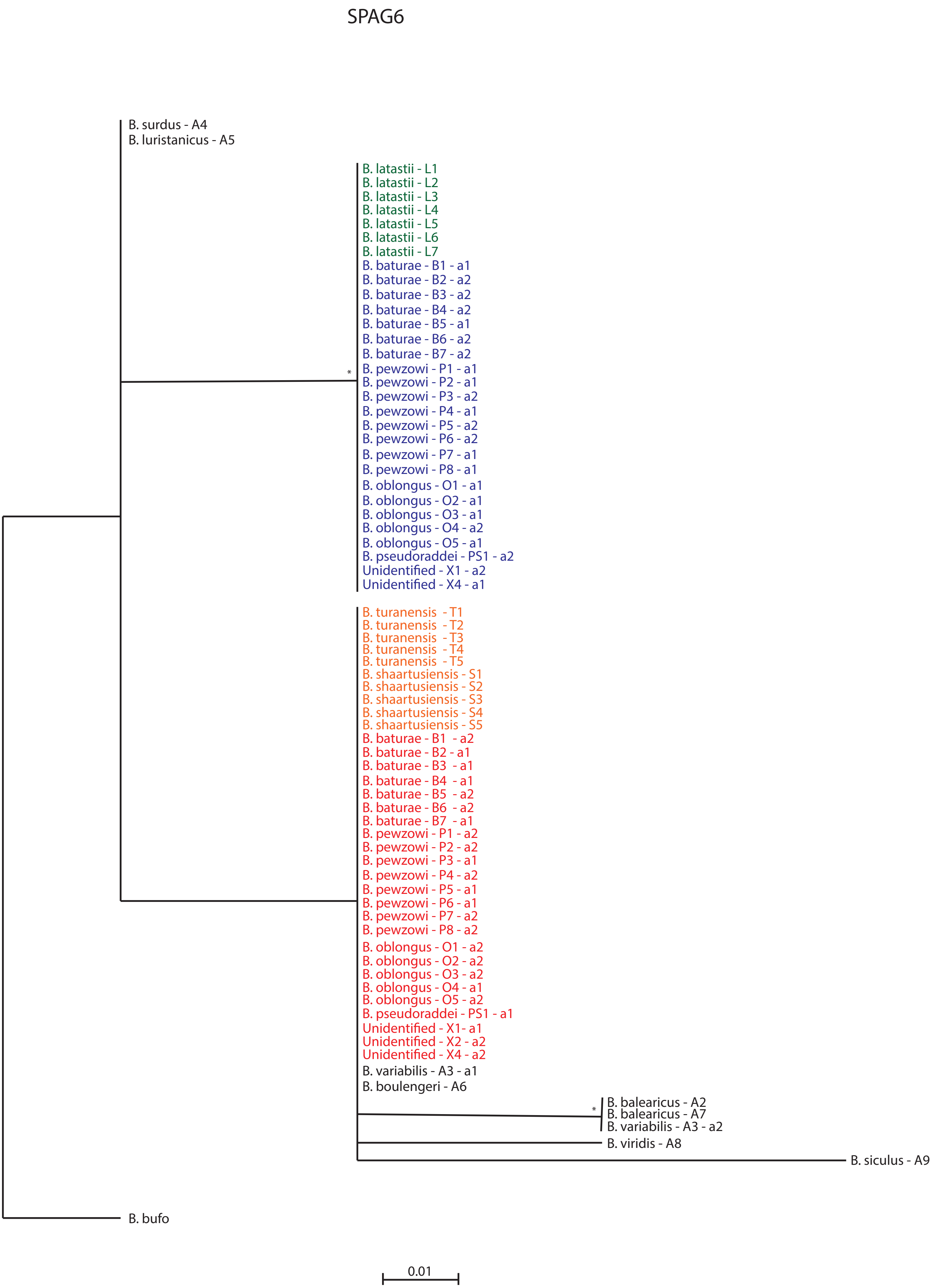
SPAG6
B. surdus - A4
B. luristanicus - A5
*
B. latastii - L1
B. latastii - L2
B. latastii - L3
B. latastii - L4
B. latastii - L5
B. latastii - L6
B. latastii - L7
B. baturae - B1 - a1
B. baturae - B2 - a2
B. baturae - B3 - a2
B. baturae - B4 - a2
B. baturae - B5 - a1
B. baturae - B6 - a2
B. baturae - B7 - a2
B. pewzowi - P1 - a1
B. pewzowi - P2 - a1
B. pewzowi - P3 - a2
B. pewzowi - P4 - a1
B. pewzowi - P5 - a2
B. pewzowi - P6 - a2
B. pewzowi - P7 - a1
B. pewzowi - P8 - a1
B. oblongus - O1 - a1
B. oblongus - O2 - a1
B. oblongus - O3 - a1
B. oblongus - O4 - a2
B. oblongus - O5 - a1
B. pseudoraddei - PS1 - a2
Unidentified - X1 - a2
Unidentified - X4 - a1
B. turanensis - T1
B. turanensis - T2
B. turanensis - T3
B. turanensis - T4
B. turanensis - T5
B. shaartusiensis - S1
B. shaartusiensis - S2
B. shaartusiensis - S3
B. shaartusiensis - S4
B. shaartusiensis - S5
B. baturae - B1 - a2
B. baturae - B2 - a1
B. baturae - B3 - a1
B. baturae - B4 - a1
B. baturae - B5 - a2
B. baturae - B6 - a2
B. baturae - B7 - a1
B. pewzowi - P1 - a2
B. pewzowi - P2 - a2
B. pewzowi - P3 - a1
B. pewzowi - P4 - a2
B. pewzowi - P5 - a1
B. pewzowi - P6 - a1
B. pewzowi - P7 - a2
B. pewzowi - P8 - a2
B. oblongus - O1 - a2
B. oblongus - O2 - a2
B. oblongus - O3 - a2
B. oblongus - O4 - a1
B. oblongus - O5 - a2
B. pseudoraddei - PS1 - a1
Unidentified - X1- a1
Unidentified - X2 - a2
Unidentified - X4 - a2
B. variabilis - A3 - a1
B. boulengeri - A6
*
B. balearicus - A2
B. balearicus - A7
B. variabilis - A3 - a2
B. viridis - A8
B. siculus - A9
B. bufo
0.01

VLDLR

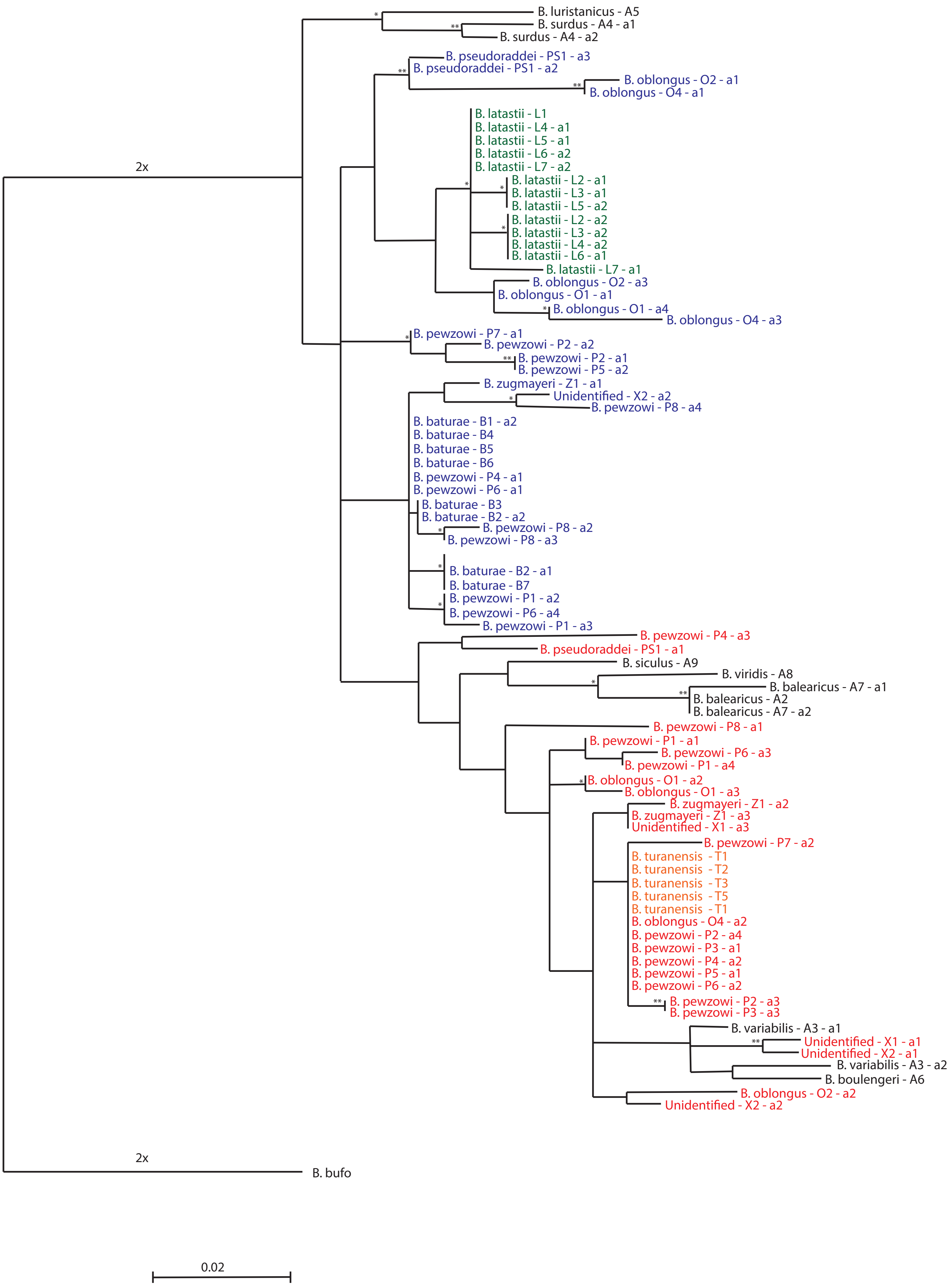

B. luristanicus - A5
B. surdus - A4 - a1
B. surdus - A4 - a2
B. pseudoraddei - PS1 - a3
B. pseudoraddei - PS1 - a2
B. oblongus - O2 - a1
B. oblongus - O4 - a1
B. latastii - L1
B. latastii - L4 - a1
B. latastii - L5 - a1
B. latastii - L6 - a2
B. latastii - L7 - a2
B. latastii - L2 - a1
B. latastii - L3 - a1
B. latastii - L5 - a2
B. latastii - L2 - a2
B. latastii - L3 - a2
B. latastii - L4 - a2
B. latastii - L6 - a1
B. latastii - L7 - a1
B. oblongus - O2 - a3
B. oblongus - O1 - a1
B. oblongus - O1 - a4
B. oblongus - O4 - a3
2x
B. pewzowi - P7 - a1
B. pewzowi - P2 - a2
B. pewzowi - P2 - a1
B. pewzowi - P5 - a2
B. zugmayeri - Z1 - a1
Unidentified - X2 - a2
B. pewzowi - P8 - a4
B. baturae - B1 - a2
B. baturae - B4
B. baturae - B5
B. baturae - B6
B. pewzowi - P4 - a1
B. pewzowi - P6 - a1
B. baturae - B3
B. baturae - B2 - a2
B. pewzowi - P8 - a2
B. pewzowi - P8 - a3
B. baturae - B2 - a1
B. baturae - B7
B. pewzowi - P1 - a2
B. pewzowi - P6 - a4
B. pewzowi - P1 - a3
B. pewzowi - P4 - a3
B. pseudoraddei - PS1 - a1
B. siculus - A9
B. viridis - A8
B. balearicus - A7 - a1
B. balearicus - A2
B. balearicus - A7 - a2
B. pewzowi - P8 - a1
B. pewzowi - P1 - a1
B. pewzowi - P6 - a3
B. pewzowi - P1 - a4
B. oblongus - O1 - a2
B. oblongus - O1 - a3
B. zugmayeri - Z1 - a2
B. zugmayeri - Z1 - a3
Unidentified - X1 - a3
B. pewzowi - P7 - a2
B. turanensis - T1
B. turanensis - T2
B. turanensis - T3
B. turanensis - T5
B. turanensis - T1
B. oblongus - O4 - a2
B. pewzowi - P2 - a4
B. pewzowi - P3 - a1
B. pewzowi - P4 - a2
B. pewzowi - P5 - a1
B. pewzowi - P6 - a2
B. pewzowi - P2 - a3
B. pewzowi - P3 - a3
B. variabilis - A3 - a1
Unidentified - X1 - a1
Unidentified - X2 - a1
B. variabilis - A3 - a2
B. boulengeri - A6
B. oblongus - O2 - a2
Unidentified - X2 - a2
2x
B. bufo
0.02
